# Adhesive bioactive materials in ocular applications: Toward smart, regenerative, and minimally invasive therapies

**DOI:** 10.1016/j.bioactmat.2025.12.004

**Published:** 2025-12-12

**Authors:** Yifei Niu, Saiqun Li, Fei Yu, Xuan Zhao, Jin Yuan

**Affiliations:** a State Key Laboratory of Ophthalmology, Zhongshan Ophthalmic Center, Sun Yat-sen University, Guangdong Provincial Key Laboratory of Ophthalmology and Visual Science, Guangzhou, Guangdong Provincial Clinical Research Center for Ocular Diseases 510060, China; b Guangdong Provincial Key Laboratory of Malignant Tumor Epigenetics and Gene Regulation, Sun Yat-Sen Memorial Hospital, Sun Yat-Sen University, 107 Yanjiang Road, Guangzhou, 510120, China; c Beijing Tongren Eye Center, Beijing Tongren Hospital, Capital Medical University, Beijing Key Laboratory of Ophthalmology & Visual Sciences, Beijing, 100730, China

**Keywords:** Adhesive bioactive materials, Ocular applications, Wet tissue adhesion, Corneal repair, Retinal repair

## Abstract

Ocular adhesive bioactive materials represent a paradigm shift in ophthalmic surgery and tissue repair, offering sutureless solutions with enhanced biocompatibility, reduced complications, and improved clinical outcomes. Designed to function as sealants, defect fillers, and delivery vehicles for drugs or cells, these materials must meet the stringent physiological and optical demands of the ocular environment. They are typically classified by anatomical application (ocular surface vs. fundus) and material origin (natural vs. synthetic), and rely on diverse crosslinking strategies to achieve tailored mechanical and adhesive properties. Current design approaches increasingly embrace biomimetic principles—aiming to replicate the structural and functional characteristics of native ocular tissues—to improve integration and therapeutic effectiveness. Moreover, the combination of adhesive materials with regenerative therapies such as stem cells, and exosomes extends their potential from simple structural support to active tissue regeneration. This review provides a comprehensive synthesis of ocular adhesive bioactive materials, outlines major design strategies and applications, and highlights future directions toward personalized and programmable regenerative platforms capable of addressing complex ophthalmic challenges.

## Highlights

• Integrates adhesion mechanisms, crosslinking strategies, and ocular applications.
• Emphasizes repair-synchronized biodegradation and optical/photochemical safety.
• Expands from sealants to regenerative scaffolds with drug/cell delivery functions.
• Summarizes advances in wet adhesion, hierarchical networks, and microstructuring
• Outlines future directions: personalized printing, exosome/stem cell integration.

## Graphical abstract

**Figure:**
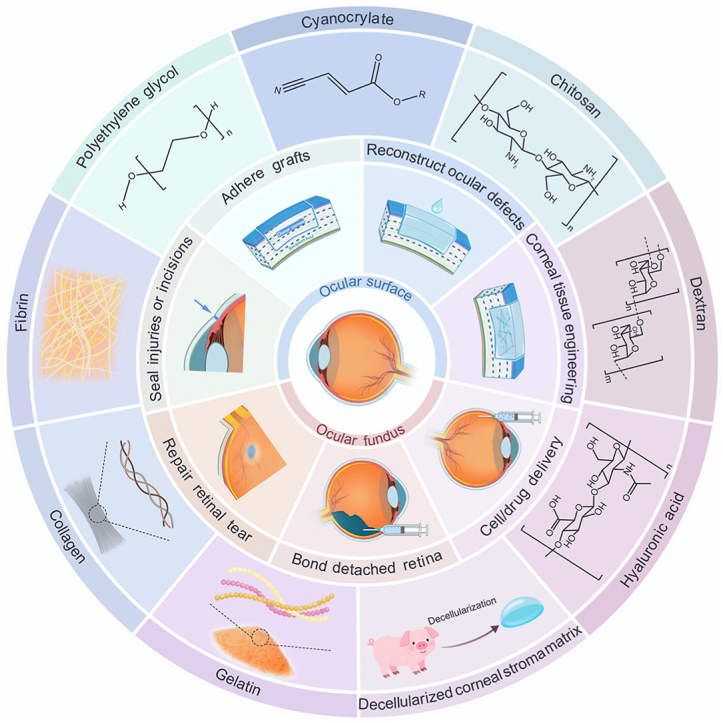

## 1 Introduction

In ophthalmic clinical practice, conventional surgical techniques and repair strategies face multiple technical limitations. First, nearly all ocular surgeries require corneal or scleral incisions, which are typically closed with sutures. However, this approach can lead to complications such as astigmatism, inflammation, infection, and secondary neovascularization, often resulting in suboptimal wound healing [[1], [2], [3]]. Second, penetrating keratoplasty (PKP) remains the primary treatment for corneal defects, yet it is constrained by donor shortages, infection risks, and immune rejection, demanding advanced surgical skills for precise suturing and stromal alignment. For posterior segment diseases, standard procedures such as pars plana vitrectomy (PPV) and scleral buckling (SB)—commonly used for rhegmatogenous retinal detachment (RRD), macular holes (MHs), and proliferative vitreoretinopathy (PVR)—exhibit notable drawbacks, including mandatory postoperative positioning, visual field defects, and PVR recurrence following vitrectomy [4,5]. These therapeutic shortcomings stem from three key challenges: (1) biocompatibility issues (chronic inflammation induced by sutures or implants), (2) anatomical disruption (such as suture tension altering corneal biomechanics, SB affecting axial length), and (3) incomplete functional recovery (failure to suppress pathological cell migration and fibrotic tissue regeneration). Globally, tens of thousands of patients require repeat surgeries or suffer irreversible vision loss due to these limitations, underscoring the urgent need for a paradigm shift in ophthalmic intervention. These limitations motivate the development of alternatives capable of rapid tissue closure with minimal trauma.

Adhesive bioactive materials have emerged as a promising solution, with their earliest applications dating back to mid-20th-century innovations. Katzin et al. (1946) demonstrated fibrin-based adhesives for rabbit corneal incisions, laying the foundation for subsequent studies [6]. Since then, fibrin-based and cyanoacrylate-derived adhesives are the most widely used adhesive bioactive materials in ophthalmology. Fibrin-based adhesives exhibit excellent biocompatibility but are limited by weak adhesion, short functional duration, and high costs [[7], [8], [9], [10]]. Conversely, cyanoacrylates offer superior bonding strength but often trigger inflammation and foreign-body reactions [[11], [12], [13], [14], [15], [16], [17], [18]].

Compared with adhesives used in other surgical fields, ophthalmic adhesives must meet unique requirements. Optical transparency, refractive index compatibility, and resistance to swelling are essential to avoid visual distortion. Adhesion must be maintained in a continuously hydrated environment subjected to dynamic shear forces from blinking, tear flow, and intraocular pressure fluctuations. Ophthalmic materials must combine conformability and flexibility for thin ocular tissues with toughness to prevent delamination. Their degradation profiles must synchronize with tissue healing kinetics—short-term for epithelial closure, longer for stromal remodeling, and extended stability for retinal reinforcement. Repair-synchronized biodegradation requires coordinated tuning of crosslinking chemistry, network architecture, and degradable motifs so that adhesion strength, modulus, and mass loss track tissue-specific healing. Cytocompatibility and photochemical safety, particularly in posterior-segment applications, remain non-negotiable design imperatives. These constraints define the design space for ocular adhesive bioactive materials.

As our understanding of ocular wound healing advanced, adhesive systems were redesigned to not merely oppose healing but to actively modulate and facilitate it. Initially introduced as rapid sealants to close incisions and prevent fluid or gas leakage, ocular adhesives have since evolved into multifunctional platforms capable of bonding tissues, supporting structural reconstruction, serving as tissue-engineering scaffolds, and delivering bioactive agents. This trajectory reflects a broader paradigm shift in ophthalmic biomaterials—from providing simple mechanical closure toward enabling programmed tissue repair and functional recovery.

Current research highlights several frontiers in ocular adhesive bioactive materials, including the development of wet-state adhesion strategies, microstructural customization to promote tissue repair, and the integration of therapeutic delivery functions. To address the eye's hydrated environment, researchers have developed wet-state adhesion strategies that enhance interfacial bonding under physiological conditions. Architected networks and anisotropic fiber alignment have been employed to preserve corneal cell phenotype, minimize scarring, and facilitate stromal gap filling and lamellar reconstruction. Moreover, adhesive bioactive materials increasingly function as carriers for growth factors and exosomes, thereby accelerating epithelialization, suppressing fibrosis, and preserving neural integrity. In the relatively static posterior segment, where adhesive demands are lower but drug delivery remains inefficient, adhesives are designed not only to seal retinal breaks and reinforce chorioretinal adhesion but also to enable sustained intraocular delivery of therapeutics—providing immediate structural stabilization alongside long-term disease modification.

This review advances the field by systematically summarizing recent developments in adhesive bioactive materials for ocular applications, with a framework encompassing adhesion mechanisms, crosslinking strategies, and functional properties. Looking forward, we highlight translational opportunities through integration with emerging technologies such as 3D printing for personalized fabrication, exosome- and stem cell-based regenerative factors, and stimuli-responsive release systems, while also emphasizing key challenges including durable wet adhesion and long-term biosafety. Together, these perspectives outline a roadmap for translating ocular adhesive bioactive materials into reliable clinical use.

## 2 Adhesion mechanisms of ocular adhesive bioactive materials

### 2.1 Design framework: interfacial linkages and energy dissipation

**Fig. 1:**
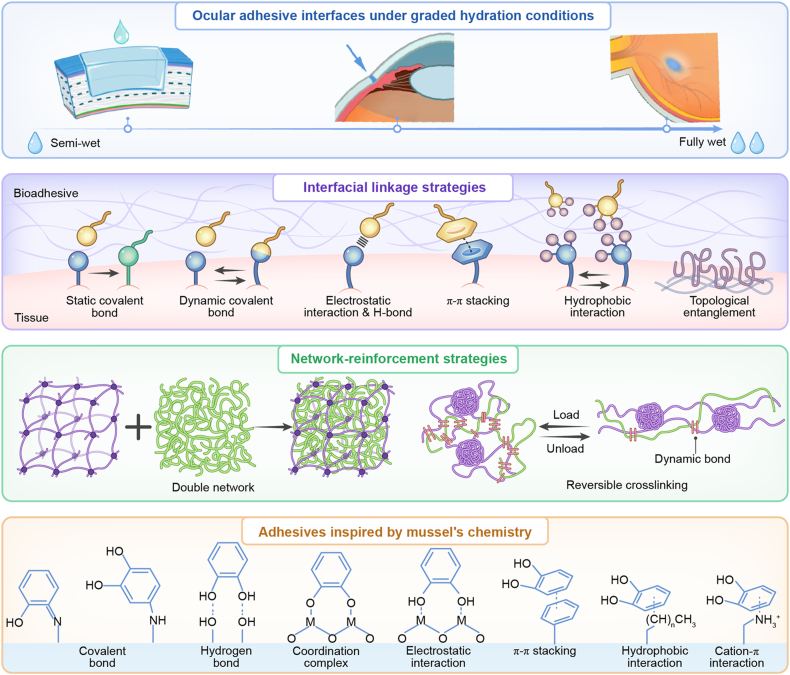
Ocular wet-adhesion mechanisms and designs.

Adhesive strength is a critical parameter for bioactive adhesive materials, making an in-depth discussion of adhesion mechanisms essential. Wu and Zhao decomposed the concept of “tissue adhesion mechanics” into two primary design levers: interfacial linkages and energy dissipation [19]. The former addresses the ability of adhesives to establish stable bonding with tissues, whereas the latter concerns the capacity to endure dynamic physiological environments once adhesion has been achieved. Additionally, tissues themselves possess viscoelastic and porous properties, which contribute to external energy dissipation and interact with the adhesive's energy-dissipating network. However, the contribution of tissues is typically limited compared with that of the adhesive bulk. Therefore, enhancing the toughness and energy-dissipation capability of the adhesive matrix is the principal strategy for improving durability. For this reason, high-performance systems frequently employ double-network or composite structures, reversible crosslinking, and swelling control to enhance the overall energy dissipation threshold while minimizing warping and interfacial delamination under moist conditions [[20], [21], [22]] (Fig. 1).

### 2.2 Interfacial adhesion modes at the tissue–adhesive interface

The interfacial adhesion mechanisms can be broadly categorized into intermolecular interactions (e.g., covalent bonds, hydrogen bonding, electrostatic forces, hydrophobic interactions, and π–π/cation–π interactions), topological entanglement (polymer chain penetration/diffusion leading to “molecular suturing”) [21], mechanical interlocking (embedding within micro-rough or porous surfaces), contact splitting (multiple micro-contacts that collectively increase critical force), and adsorption/negative pressure (suction-cup-like liquid expulsion and pressure differentials) [19]. Most currently used ocular adhesives rely primarily on intermolecular interactions. This approach offers simplicity, rapid interfacial bonding, and sufficient adhesion strength for ocular applications. Nevertheless, we recommend a synergistic strategy that combines intermolecular bonding with structural anchoring to address the challenges posed by the moist and dynamic ocular environment.

### 2.3 Bulk energy-dissipating networks and swelling control

Since interfacial linkages alone are insufficient to withstand continuous tissue deformation and fatigue, bulk energy-dissipating networks are indispensable. Common strategies include introducing chain scission and reversible crosslinking (e.g., hydrogen bonding, metal coordination, or dynamic covalent bonds) as energy traps [23], employing multifunctional crosslinking and composite/double networks to disperse stress [20]. Multifunctional crosslinking creates bonds with distinct strengths and kinetics, redistributing loads across time and length scales. Composite or double networks pair a brittle network with an elastic matrix, localizing damage and dissipating energy while the matrix preserves overall integrity. Furthermore, swelling of adhesives in wet environments alters both network mechanics and interfacial stress distribution. Formulation and structural constraints are thus necessary to maintain a balance between adhesion and toughness.

### 2.4 Wet-adhesion strategies in ocular environments

In clinical practice, adhesives operate under far from ideal conditions, facing three simultaneous challenges: contamination of reactive groups by body fluids, disruption of molecular contact by surface hydration layers, and continuous tissue motion imposing tensile, shear, and fatigue stresses. Effective wet adhesion requires either displacing interfacial water or forming stable interactions in aqueous environments. One drainage-based strategy involves applying adhesives in powder form, which dissolve in surface moisture at the target site to form hydrogels while simultaneously removing interfacial water, enabling rapid adhesion within seconds [24]. However, this method is unsuitable for ocular applications because it produces rough surfaces, uneven adhesive thickness, and limited resistance to contamination. We therefore propose redesigning such powders into pre-formed patches, which integrate the “dehydration–adhesion” process into a single step, simplifying application. Another drainage strategy coats patches with a hydrophobic liquid immiscible with body fluids, serving as a dynamic barrier to repel water or blood. Upon mechanical compression at the target site, the hydrophobic layer is expelled, allowing direct tissue–adhesive contact and bond formation [25]. While effective in enhancing resistance to contamination in wet ocular environments, this approach necessitates careful evaluation of biocompatibility, migration, and long-term retention of the hydrophobic phase. Inspired by the microstructures of octopus suckers, patterned surface designs incorporating microgrooves have also been proposed. These patterns enable rapid fluid drainage, increase effective contact area, and synergize with frictional and suction forces to improve adhesion [26]. This method offers advantages for reversible adhesion on rough substrates, though manufacturing complexity and geometric stability remain major obstacles.

Another strategy for achieving wet adhesion is to design adhesives that function effectively in aqueous environments. Mussel-inspired polyphenol/catechol chemistry exemplifies this approach. By mimicking mussel foot proteins, researchers have developed catechol (DOPA/dopamine)-containing side chains that bond to tissues via hydrogen bonding, π–π stacking, or metal coordination; undergo oxidative covalent coupling with amines/thiols; and construct reversible energy-dissipation networks through metal–catechol coordination [27]. This multi-modal bonding mechanism converts strong adhesion into durable adhesion while offering self-healing and energy dissipation. Beyond mussel-inspired chemistry, gecko-inspired setae/spatulae structures provide another bioinspired strategy. These structures divide large contact areas into numerous nanoscale contacts, enhancing adaptability and overall adhesion strength via van der Waals interactions [28]. Synthetic nanocolumns mimicking this strategy increase surface area [29] and can be chemically functionalized to promote covalent crosslinking with tissues, thereby stabilizing adhesion. To address wet conditions, mushroom-shaped tips have been further incorporated, significantly improving adhesion stability in aqueous environments [30]. Illustrative schematics for the adhesion mechanisms summarized above can be found in the figures of the review by S.J. Wu and Zhao [19].

### 2.5 Translational barriers and design opportunities for ophthalmic wet adhesives

To date, wet-adhesion strategies in ophthalmic applications remain limited. Most currently used ocular adhesives rely primarily on intermolecular interactions, with dynamic covalent linkages and catechol-based (mussel-inspired) chemistries providing the clearest evidence base. Other interfacial/structural mechanisms summarized above—such as topological entanglement, mechanical interlocking, and contact splitting—are promising but under-tested in the eye and warrant targeted ophthalmic validation.

Gecko-inspired setae/spatulae structures and octopus-sucker-inspired microstructures have seen little clinical translation in ophthalmology. The principal barrier is not inadequate intrinsic adhesion but a structural mismatch between their operating principles and the optical, mechanical, and fluidic constraints of the ocular surface. Micro- and nano-periodic features can introduce light scattering and diffraction that degrade visual quality [31]. The tear film and epithelial glycocalyx reduce the effective contact area and hinder intimate interfacial coupling [32]. Sucker-based concepts depend on fluid evacuation and edge sealing to maintain negative pressure, yet continuous tear replenishment and epithelial microporosity make a sustained vacuum difficult. In addition, adsorption or suction-based approaches impose strict intraoperative compression and placement requirements that are incompatible with rapid, standardized procedures. Future translation may be facilitated by hybrid designs that graft catechol, multidentate hydrogen-bonding, or dynamic covalent motifs onto the contact surface, combined with spatially selective patterning—excluding microstructures over the pupillary axis and confining them to a peripheral annulus—to preserve optical quality while retaining drainage or anchoring functions.

## 3 Classification of ocular adhesive bioactive materials

**Fig. 2:**
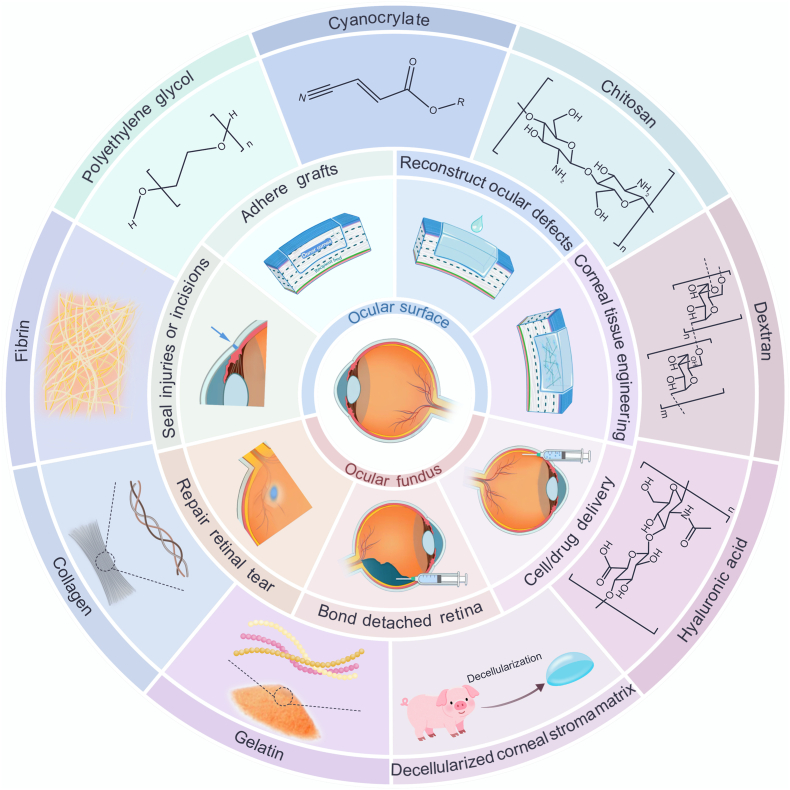
Ocular adhesive bioactive materials categorized by application type and material origin.

Recent advances in biomaterial engineering have led to a diverse array of ocular adhesive bioactive materials with improved performance. See Supplementary Table S1 for a consolidated summary of all material characteristics discussed in the text. From an anatomical perspective, they are further classified according to their application in the ocular surface or the ocular fundus (Fig. 2). Based on material origin, ocular adhesive bioactive materials can be categorized into naturally derived and synthetic materials (Table 1). Naturally derived bioactive materials, sourced from biological polymers, offer advantages such as superior biocompatibility, controlled biodegradability, reduced immunogenicity, and the potential to stimulate endogenous repair mechanisms. In contrast, synthetic polymers exhibit well-defined chemical compositions, ensuring reproducible fabrication processes and precise control over material properties. Regarding crosslinking mechanisms, these materials primarily employ physical or chemical crosslinking strategies. Physical crosslinking relies on non-covalent interactions (e.g., hydrogen bonds, hydrophobic forces, electrostatic attractions) to form reversible networks, enabling dynamic responsiveness to environmental stimuli [33,34]. However, such networks exhibit lower mechanical strength and are susceptible to degradation under physiological stresses [35] (e.g., eyelid movement or tear flow). Chemical crosslinking, initiated upon component mixing, generates covalent networks with high mechanical stability and tunable properties via crosslink density modulation. However, this approach often involves complex protocols, stringent reaction conditions, and potential cytotoxicity from residual crosslinking agents [36]. Photo-crosslinking, a subset of chemical crosslinking, utilizes ultraviolet (UV)/visible light to trigger polymerization of photosensitive groups, enabling in situ hydrogel formation. Although UV-initiated systems enable minimally invasive application, UV exposure poses tissue-damage risks and limited penetration constrains deep-tissue use [37]. Certain photoinitiators enable visible light-induced polymerization as an alternative to UV light initiation [38]. However, the potential adverse effects of visible light exposure parameters (including intensity and duration) on ocular tissues must be carefully considered. In terms of fabrication, materials are either pre-formed or designed for in situ formation. In situ systems, usually delivered via injection, offer minimally invasive application and superior adaptability to irregular defects [39,40]. Nevertheless, their mechanical strength is often compromised, and crosslinking efficiency may be hindered by physiological conditions. Pre-formed materials, conversely, provide consistent mechanical properties, batch-to-batch uniformity, and long-term stability but lack the flexibility of in situ systems [41]. Thus, in situ materials are preferred for short-term, defect-specific applications, whereas pre-formed variants suit long-term implants.

**Table 1:** Comparison of characteristics: Naturally derived vs. Synthetic bioactive materials.

Properties	Naturally derived bioactive materials	Synthetic bioactive materials
Biocompatibility	Higher	Lower
Degradability	Naturally biodegradable	Controlled degradation via crosslinking strategies
Mechanical Properties	Limited, often needing reinforcement	Able to be precisely controlled
Immune Response	Lower	Higher
Cost	More expensive	Cheaper
Ethical Issues	Yes	No

In this review, ocular adhesive bioactive materials are introduced in sequence from the ocular surface to the ocular fundus, classified according to their principal components.

## 4 Adhesive bioactive materials for the ocular surface

Most ocular surgeries rely on sutures to close incisions, yet this approach is time-consuming, technically demanding, and associated with complications such as infection, corneal neovascularization, and astigmatism [42]. Additionally, the removal of sutures at a later stage may lead to suture-related complications [43]. Corneal transplantation, as the traditional treatment for corneal perforation and corneal leukoplakia, is constrained by limitations such as donor cornea shortages, immune rejection, and the need for advanced surgical expertise [[44], [45], [46]]. To address these clinical challenges, numerous novel adhesive bioactive materials have been developed as alternatives to corneal transplantation, aimed at repairing corneal stromal defects (Table 2). Since the ocular surface is a semi-wet, dynamic interface, adhesive materials designed for this environment must exhibit rapid curing (within seconds to minutes), strong wet adhesion and resistance to fatigue while ensuring optical transparency and refraction compatibility. Their degradation must synchronize with epithelial closure and stromal remodeling, which typically occurs on a short-to medium-term scale (from days to weeks). Additionally, adhesive materials must maintain low friction and smooth surfaces to avoid foreign-body sensation or neovascularization. These materials offer significant clinical potential by reducing surgery time, eliminating the need for sutures, promoting tissue regeneration, and decreasing the risk of rejection and infection.

**Table 2:** Characteristics and evidence overview of ocular surface adhesive bioactive materials.

Material Type	Adhesion/Interface Strategy	Curing/Triggering Method	Typical Gelation Time	Representative Properties			Observed Adhesion Time	Main Advantages	Main Limitations	Evidence Level
				Burst Pressure	Young's modulus	Transparency				
Fibrin glue [62]	Coagulation mimicry: covalent/hydrogen/electrostatic bonds with collagen	Two-component mixing	Seconds–minutes	∼91 ± 27 mmHg	/	Good	Days–weeks	Good biocompatibility, promotes healing	Adhesion strength and functional duration are limited; batch variability, potential immunogenic/contamination risks	Animal/clinical mix
Collagen-based [65,69,70,73]	Schiff base/SPAAC/Thiol-Maleimide/Bi-network; Topological infiltration	Chemical/Light crosslinking	Minutes	∼63.5–71.1 mmHg	/	70–99 %	Days–weeks (up to 28 days)	Low immunogenicity, promotes regeneration, transparent	Initial mechanical weakness; light/chemical residual safety concerns	In vivo/early clinical
Gelatin-based [21,[93], [94], [95], [96], [97], [98], [99],^101^,^103^]	Dopamine/catechol, bi-network, HA/SF composites	Visible light/UV (mostly visible light); Dual-trigger	Seconds–minutes	∼51–610 mmHg	∼27–300 kPa	80–100 %	2–8 weeks (up to 6 months)	Strong wet adhesion, transparent, injectable/in-situ gel	Anti-swelling/long-term mechanical properties need strengthening; light-chemical safety and discoloration need control	Animal/functional validation
Decellularized Corneal Matrix-based [20,109,[112], [113], [114]]	Near-native ECM molecular/microstructure adhesion + secondary crosslinking reinforcement	Visible light/chemical crosslinking; Bi-network	Minutes	∼22.7–542 mmHg	∼607 kPa	80–90 %	2–24 weeks (up to 6 months)	Near-native structure and optics, promotes regeneration	Structural anisotropy weakened, source & standardization	Animal/translation
HA-based [22,38,124,125]	Host-guest supramolecular/catechol/dual-trigger	Light/chemical/thermal-light dual-trigger	Within 90 s	∼70–1200 mmHg	/	80–99 %	Days–weeks (some longer)	High compatibility, anti-inflammatory/oxidative, reversible/shear thinning	Quick clearance requires crosslinking; certain crosslinkers/initiators safety concerns	Animal/organ
Dextran-based [128]	Schiff base + multi-mechanism synergy (ion/radical)	Chemical + light + ion triple	Seconds–minutes	/	∼290 kPa	/	≤90 days	Rapid gelation, transparent, tunable degradation	Dynamic bonds may reverse too strongly under high hydration/shear	Animal
Chitosan-based [134,135,137,138]	Cationic + covalent/light/laser welding	Light/laser/chemical	Seconds–minutes	∼229 ± 54 mmHg	∼78.5 ± 13.0 kPa	74–99 %	2–12 weeks	Antibacterial, promotes epithelialization, drug/exosome delivery	Sensitive to pH/ions; residual toxicity from traditional crosslinkers	Animal/small sample
Cyanoacrylate [281]	Rapid anionic polymerization to form solid film	In-situ polymerization (wet surface)	Seconds	∼400 mmHg	/	/	1–4 weeks (median ∼29 days)	Rapid sealing, high strength	Poor biocompatibility, heat/exothermic/rough opacity, toxic by-products	Extensive clinical observation
PEG-based [[155], [156], [157], [158], [159], [160], [161], [162], [163], [164], [165], [166],[168], [169], [170],^173^,^174^]	NHS/amino/amide covalent; collagen/polysaccharide composites	Multi-component instant reaction/light	≤30 s (ReSure®)	∼37–115 mmHg (ReSure®)	∼30–160 kPa	20–100 %	Days–weeks	Transparent, inert, modifiable, clinically accessible	Short application window; limited durability under high hydration/shear	Animal/clinical

**Abbreviations**: SPAAC: strain-promoted azide–alkyne cycloaddition, HA: hyaluronic acid, SF: silk fibroin, UV: ultraviolet, ECM: extracellular matrix, PEG: polyethylene glycol, NHS: N-hydroxysuccinimide.

### 4.1 Protein-based bioactive materials for the ocular surface

Protein-based adhesive bioactive materials, including those derived from blood, collagen, gelatin, and decellularized corneal stroma matrix, demonstrate mechanical properties that closely resemble those of the corneal stroma and exhibit excellent tissue compatibility. These attributes make them highly suitable for ocular surface reconstruction. To address limitations related to insufficient mechanical strength and weak stability, recent research efforts have focused on continuous improvements through the design of hierarchical network structures and precise control over the crosslinking process [20]. In corneal tissue engineering, hierarchical network structures are multi-scale ordered arrangements that replicate the native stromal architecture. This design ensures clarity, strength, and long-term stability, making it essential for functional artificial corneal substitutes.

#### 4.1.1 Blood-derived bioactive materials for the ocular surface

The blood-derived bioactive materials proposed in this review encompass fibrin-based, platelet lysate-based, and serum albumin-based bioactive materials.

Fibrin-based bioactive materials, being the earliest naturally derived bioactive materials, entered commercial markets in the 1970s and received initial Food and Drug Administration (FDA) approval as an adjunct to hemostasis in general and cardiovascular surgeries [47]. In the United States, they received approval for medical use in 2003, and in the European Union (EU), they were approved in 2008. Some of the products that have been commercialized include Artiss, Evicel, and Tisseel [[48], [49], [50]]. The two-component system of fibrin-based bioactive materials comprises: (1) a sealant protein solution containing human fibrinogen, plasminogen, fibronectin, and coagulation factor XIII in a solution of bovine aprotinin; and (2) a sealant setting solution containing reconstituted human thrombin in a solution of calcium chloride [51]. Fibrin-based bioactive materials mimic the coagulation process in their adhesion mechanism, resulting in the formation of a biocompatible plasma clot akin to the natural fibrin matrix. Upon mixing these components, thrombin converts fibrinogen to fibrin, and factor XIII facilitates cross-linking of fibrin monomers, leading to fibrin clot formation. Fibrin and fibronectin form cross-links with collagen in tissues, creating covalent, hydrogen, and electrostatic bonds, thereby imparting high adhesion of fibrin-based bioactive materials to the collagen-rich ocular tissue [52].

Due to their biocompatibility and biodegradability, fibrin-based bioactive materials can effectively cover larger surface areas and facilitate corneal healing by employing superficial coverings like amniotic or conjunctival membranes [10]. Additionally, they pose a lower risk of complications such as corneal neovascularization, secondary corneal ulcers, and impaired healing, while also offering enhanced patient comfort [53]. Reports suggest that fibrin-based bioactive materials have been utilized in securing: (1) corneal grafts in lamellar penetrating scleral keratoplasty, (2) corneal flaps in laser-assisted in situ keratomileusis (LASIK), (3) conjunctival flaps in pterygium surgery and forniceal reconstruction, (4) the closure of corneal perforations and descemetoceles, and (5) amniotic membrane transplantation and conjunctival autotransplantation [8,[54], [55], [56], [57]].

However, bioactive fibrin-based materials also present several challenges. First, fibrin clots often lack sufficient adhesive strength and duration, dissolving within a few days to weeks. This dissolution can lead to unstable wound healing and graft detachment. Second, immune responses to clotting factors and thrombin may trigger systemic reactions, including allergies and coagulopathies [58]. Additionally, human blood products contaminated with viruses could potentially transmit diseases such as hepatitis and acquired immunodeficiency syndrome (AIDS), which remains a theoretical concern [59,60]. Furthermore, improper application of fibrin glue can result in its accumulation on grafts, forming a physical barrier that delays epithelial cell migration [61]. This issue imposes higher demands on surgical techniques and undermines the advantages of shorter operative and healing times.

Human platelet lysate (hPL)-based bioactive materials are derived from fibrin-based bioactive materials augmented with human platelet lysate. The hPL-based bioactive materials comprise components akin to those found in fibrin glue, but with reduced concentrations of fibrinogen and thrombin, more closely resembling physiological levels, thus easing both the manufacturing process and the restoration of visual function [62]. Moreover, mediums containing platelet lysates have been found to inhibit fibrosis of corneal stromal cells, potentially conferring an added advantage to the utilization of hPL-based bioactive materials [63].

Bovine serum albumin-based bioactive material represents a novel photosensitive tissue adhesive capable of rapid solidification under laser irradiation, forming a gel characterized by high strength, excellent biocompatibility, and moderate degradability. This rapid gelation arises from the intrinsic thermal denaturation of albumin molecules: upon localized heating by laser energy, the protein's tertiary structure unfolds, exposing hydrophobic domains and reactive thiol groups that subsequently undergo intermolecular crosslinking, thereby generating a stable three-dimensional gel network. The CO_2_ laser, whose wavelength is strongly absorbed by water, provides highly localized heating, enabling precise control of temperature in the optimal range (∼60–70 °C) for albumin denaturation without inducing extensive tissue damage. Tal et al. demonstrated successful repair of large perforating incisions in porcine corneas utilizing temperature-controlled laser soldering with bovine albumin, proving its superiority over conventional suturing techniques by achieving faster closure, reduced inflammation, and improved wound integrity [64].

Blood-derived bioactive materials, such as fibrin, platelet lysate, and albumin-based materials, offer excellent biocompatibility and biodegradability, making them highly suitable for ocular surface repair. These materials are capable of covering large areas and promoting corneal healing through superficial membrane applications like amniotic or conjunctival membranes. However, their short adhesive strength and functional duration (usually lasting from several days to weeks), along with limited tolerance to wet environments and shear forces, are significant limitations. Additionally, batch variability and potential immunogenic concerns remain critical issues, particularly for materials derived from human blood. Furthermore, albumin-based adhesives require specific laser equipment for optimal activation, which limits their clinical applicability. Further work should focus on enhancing wet adhesion and mechanical reinforcement while ensuring standardization and safety.

#### 4.1.2 Collagen-based bioactive materials for the ocular surface

Collagen has emerged as a superior candidate for ocular repair bioactive materials due to its excellent biological compatibility, low immunogenicity, controllable biodegradability, and capacity to promote autologous tissue regeneration. However, collagen solubilization requires extreme pH conditions that are generally incompatible with physiological ocular environments. Moreover, its post-solubilization hyperviscosity compromises flow properties and impedes effective tissue infiltration, thereby failing to meet the essential fluidity requirements for bioadhesive applications. To address these limitations, researchers have developed collagen-based composite biomaterials through either chemical modification [65] or copolymer blending strategies [66]. These engineered systems form hierarchical networks that synergistically combine native biocompatibility with enhanced adhesive performance, demonstrating significant potential for corneal stromal defect restoration.

**Fig. 3:**
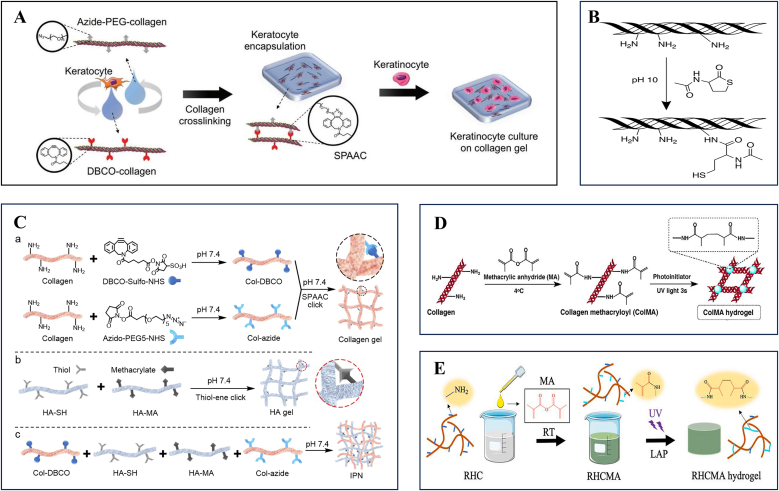
Collagen-based bioactive materials for the ocular surface. A Schematic of crosslinked collagen gel matrix formation by strain-promoted azide–alkyne cycloaddition. Azide-PEG-conjugated collagen and DBCO-conjugated collagen were mixed with keratocytes to fabricate a corneal stroma tissue substitute. Keratinocytes were seeded on the keratocyte-encapsulated collagen gel to facilitate the formation of the epithelial layer. Reprinted with permission from Ref. [39]. B Reaction scheme of the synthesis of thiol collagen. Reprinted with permission from Ref. [70]. C Formation of HA- and collagen-based interpenetrating polymer network (IPN). (a) Collagen was modified with dibenzocyclooctyne-sulfo-N-hydroxysuccinimidyl ester (DBCO-sulfo-NHS) and azido-PEG-5-N-hydroxysuccinimidyl ester (azido-PEG5-NHS) respectively. Modified collagens were then mixed and crosslinked via the bio-orthogonal strain-promoted azide-alkyne cycloaddition (SPAAC) under physiological conditions. Collagen SPAAC click gel is denoted as xCol. (b) HA thiol-ene gel (xHA) was formed by crosslinking thiolated HA (HA-SH) and methacrylated HA (HA-MA) via thiol-ene click reaction under physiological condition. (c) DBCO-modified collagen, thiolated HA, methacrylated HA, and azido-modified collagen were mixed in order to form IPN under physiological conditions. Reprinted with permission from Ref. [66]. D Scheme of methacryloylated collagen (ColMA) hydrogel formation. Reprinted with permission from Ref. [73]. E The schematic diagram of the synthesis of RHCMA hydrogels. Reprinted with permission from Ref. [65].

Amino-rich collagen is frequently combined with oxidized dextran (ODex) via Schiff-base chemistry, which is rich in aldehyde groups, to enhance mechanical strength. A Schiff base forms when an aldehyde condenses with a primary amine to yield an imine (–C=N–). These reactions proceed rapidly in water under mild, near-physiological conditions and require no added catalysts, which makes them well suited for in situ gelation of injectable precursors on delicate tissues. In the moist ocular environment, the reversible nature of simple imines can provide energy dissipation and self-healing, enabling fast gelation without external light or heat, while maintaining high chemoselectivity toward the amine groups already present on collagen [67]. Zhang et al. engineered a Col/DAD hydrogel that exhibited a twenty-fold increase in stiffness and significantly improved thermal stability compared with unmodified collagen hydrogels [68]. Building on this, Vijayan et al. incorporated gadolinium oxide nanoparticles into dextran–collagen hydrogels, which not only strengthened the mechanical performance but also imparted anti-angiogenic and anti-inflammatory properties, thereby alleviating the detrimental effects of corneal neovascularization on visual recovery [69]. Beyond exploiting Schiff base reactions, Lee et al. introduced azide-functionalized collagen through N-hydroxysuccinimide (NHS) coupling chemistry and synthesized dibenzocyclooctyne-modified collagen via strain-promoted azide-alkyne cycloaddition (SPAAC), leading to the development of in situ-forming hydrogels for repairing corneal stromal defects. These hydrogels from Lee et al.’s system facilitated corneal epithelial cell migration and proliferation on their surface while supporting stromal cell survival and spreading within the matrix (Fig. 3A) [39]. In a parallel approach, Rosenquist et al. pioneered thiol-maleimide click chemistry for injectable collagen hydrogels. Their innovation involved collagen thiolation using DL-N-acetyl homocysteine thiolactone (AHTL), which enhanced collagen solubility and reactivity (Fig. 3B). The resulting hydrogels demonstrated tunable mechanical properties comparable to natural corneal tissue and achieved tissue adhesion strength equivalent to fibrin glue [70]. Catalyst-free interpenetrating polymer networks (IPNs) have emerged as promising platforms. As a classic multiphase network strategy, IPNs refer to two or more polymer networks that are spatially interlaced yet not directly linked by covalent bonds, and are typically constructed via mutually independent curing/crosslinking pathways [71]. This topological entanglement–confinement effect enables materials to achieve higher strength/toughness, fatigue resistance, and tear resistance without sacrificing water content or transparency, while markedly reducing equilibrium swelling and swelling-induced stress concentrations. Myung's team independently crosslinked collagen and hyaluronic acid (HA) through SPAAC and mercapto-alkenyl Michael addition, respectively, forming transparent hydrogel matrices (Fig. 3C). These dual-network systems promoted corneal epithelial regeneration through controlled release of degradation products while suppressing stromal fibrosis and inflammatory responses [66,72].

Recent advancements in corneal tissue engineering have focused on developing collagen-based scaffolds that recapitulate the native cornea's structural and functional attributes. Zhang et al. optimized a UV-crosslinked methacryloylated collagen (ColMA) hydrogel for corneal defect repair (Fig. 3D) [73]. The photopolymerized network demonstrates controlled biodegradation kinetics through regulated cleavage of methacrylate bonds, enabling synchronized replacement by neo-tissue. Unlike conventional collagen matrices, this system minimizes inflammatory responses during degradation while maintaining critical mechanical integrity during the remodeling phase, thereby enhancing regenerative outcomes.

Recombinant human collagen (RHC) has emerged as a promising biomaterial for the development of collagen-based corneal adhesives. Unlike animal-derived collagens, which often suffer from batch variability, potential immunogenicity, and risk of pathogen transmission [74], RHC is produced through genetic engineering to replicate the exact amino acid sequence of native human collagen. This ensures superior biocompatibility, molecular consistency, and the possibility of tailoring structural features such as telopeptides, which are crucial for proper fibril assembly and tissue organization [75]. Moreover, RHC allows precise control over collagen type and source, thereby enabling the fabrication of adhesives that closely mimic the hierarchical architecture and transparency of the natural corneal stroma [76]. Collectively, these advantages make RHC an ethically sustainable and clinically safer alternative to animal-derived collagen for corneal repair and regeneration applications. Kong et al. engineered a RHC scaffold featuring hierarchically ordered microarchitectures through methacrylic anhydride (MA) functionalization (Fig. 3E) [65]. This biomimetic design incorporates aligned microgrooves and inverse opal nanopores, achieving biomechanical properties and light transmittance comparable to natural corneal stroma while facilitating oriented stromal cell regeneration. In a related advancement, Collagen-based materials demonstrate excellent biological compatibility, low immunogenicity, and the ability to promote autologous tissue regeneration. However, solubilization requirements for collagen and hyperviscosity post-solubilization make them less ideal for adhesive applications in ocular environments. Recent improvements in crosslinking have led to enhanced adhesive strength, but light/chemical crosslinking may still present phototoxicity or residual issues. Additionally, their mechanical strength remains insufficient without crosslinking, and degradation must be synchronized with tissue healing processes. Long-term data on stability and safety are also necessary to guide their clinical use.

#### 4.1.3 Gelatin-based bioactive materials for the ocular surface

Gelatin, a hydrolytically derived natural polymer originating from collagen, exhibits exceptional biocompatibility, rendering it a widely adopted biomaterial in tissue engineering for regenerative purposes across diverse human tissues [[77], [78], [79], [80], [81], [82], [83], [84], [85], [86]]. As a hydrolyzed derivative of collagen, gelatin retains favorable biodegradability while demonstrating enhanced solubility and reduced immunogenicity compared to its precursor. However, its low mechanical strength and susceptibility to structural degradation in high-humidity ocular environments limit its long-term stability. Additionally, conventional gelatin crosslinking relies on chemical agents such as glutaraldehyde, posing risks of residual cytotoxicity that may compromise sensitive ocular tissues.

To address these limitations, methacrylated gelatin (GelMA) has emerged as a promising modified biomaterial. GelMA introduces photocrosslinkable methacryloyl groups, enabling rapid UV or blue light-initiated curing without toxic crosslinkers [87]. By modulating GelMA concentration and substitution degrees, researchers can optimize its mechanical resilience and enzymatic resistance, ensuring structural integrity in aqueous environments [88]. Crucially, GelMA preserves critical cell-adhesive motifs (e.g., RGD sequences) inherent to gelatin [89], thereby facilitating cell migration, proliferation, and tissue regeneration. Previous studies have demonstrated that GelMA at a concentration of 5–10 % can facilitate the regeneration of the corneal stroma through minimally invasive injection or encapsulation, indicating its considerable potential for applications in tissue-engineered corneal stroma [90,91].

**Fig. 4:**
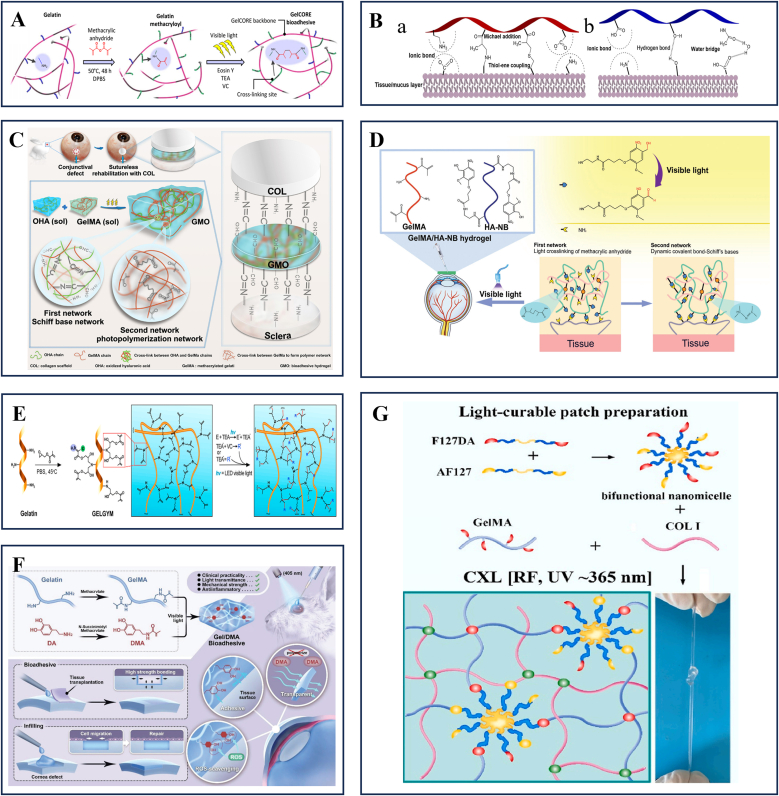
Gelatin-based bioactive materials for the ocular surface. A Schematic of the chemical reaction for GelCORE formation and photocrosslinking of the prepolymer solution with Eosin Y (photoinitiator), TEA (co-initiator), and VC (co-monomer). Reprinted with permission from Ref. [93]. B Schematic representation of the possible chemical and physical bond formations at the hydrogel/eye tissue interface with the (B) GelMA polymer chain and (C) HAGM polymer chain with the existing functional groups of the target ocular tissue. Reprinted with permission from Ref. [94]. C schematic diagram illustrating the sutureless surgery of collagen scaffold transplantation into a conjunctival defect with the help of GMO adhesive (COL, collagen scaffold; OHA, oxidized hyaluronic acid; GelMA, gelatin methacrylate; GMO, GelMA and oxidized hyaluronic acid composite bioadhesive hydrogel). Reprinted with permission from Ref. [96]. D Constituent chemical structures and schematic illustration of the phototriggered GelMA/HA-NB adhesive hydrogel and its application in large-diameter corneal defect repair. Reprinted with permission from Ref. [95]. E Schematic of chemical synthesis of GELGYM via epoxide ring-opening reaction of glycidyl methacrylate with gelatin and its crosslinking with a visible LED in the presence of E (0.05 mM), TEA 0.04 %, and VC (0.04 %) to form a 3-dimensional network of the hydrogel. Reprinted with permission from Ref. [103]. F Synthesis and application of Gel/DMA bioadhesive for sutureless ocular tissue repair. The Gel/DMA bioadhesive is developed by in situ oxidative free-radical polymerization with high light transmittance, mechanical strength, adhesion, and ROS scavenging ability. As a bioadhesive, it can reduce the inflammatory response and fibrosis formation in ocular tissue transplantation. Additionally, it also can be used as a corneal substitute to accelerate corneal re-epithelialization and wound healing. Reprinted with permission from Ref. [99]. G Fabrication and application, as well as networks illustration of light-curable adhesive corneal hydrogel patch. Reprinted with permission from Ref. [101].

Hosseini et al. demonstrated that incorporating dopamine (DA) into GelMA precursor solutions significantly enhances the hydrogel's mechanical properties and bioadhesive capabilities, particularly improving adhesion strength in aqueous environments [92]. Oxidative polymerization of DA into polydopamine (PDA) yielded PDA@GelMA hydrogels with superior elasticity, toughness, and significantly increased tensile/shear adhesive strength. Sani et al. developed GelCORE, a visible light-crosslinkable corneal adhesive that employs erythrosin B (Type II) as a co-initiator, triethanolamine (TEA), and N-vinylcaprolactam (VC) as co-monomers, polymerized via free-radical reactions to eliminate risks of UV-induced ocular phototoxicity (Fig. 4A) [93].

Chemically modified HA derivatives, known for their high viscosity, synergize with low-viscosity GelMA to improve adhesive retention [94]. Wang et al. designed a photocurable HA with nitrobenzyl groups (HA-NB)/GelMA hydrogel for large-diameter corneal defects, which, upon visible light curing, demonstrated robust mechanical properties, and strong wet-tissue adhesion (Fig. 4D) [95]. Similarly, Gholizadeh et al. developed GelPatch, comprising GelMA and glyceryl methacrylate-ylated hyaluronic acid (HAGM) with strong conjunctival/scleral adhesion, low swelling ratio, and tunable elasticity for rapid repair of ocular trauma (Fig. 4B) [94].

Liu et al. introduced a semi-interpenetrating network (sIPN) adhesive (GMO) comprising GelMA and oxidized hyaluronic acid (OHA), effectively anchoring collagen scaffolds in corneal defects and showing clinical promise for suture-free repair (Fig. 4C) [96]. Beyond HA, alternative materials like silk fibroin (SF) have been integrated with GelMA. Tutar et al. formulated a GelMA/SF (GS) composite with enhanced mechanical strength and adhesion, promoting fibroblast proliferation and attachment for corneal wound healing [97]. Owing to its abundance of aldehyde groups, ODex is also frequently incorporated into gelatin-based systems. To address the mechanical limitations of single-network hydrogels, Zhao et al. engineered a GelMA-ODex hydrogel for sutureless corneal transplantation, leveraging Schiff base reactions between GelMA and ODex as the first network, followed by visible light-initiated crosslinking to form a second network, thereby enhancing mechanical resilience and tissue adhesion [98].

Similarly, Tan et al. developed a dual covalent cross-linking hydrogel (ASO) bioadhesive, composed of acrylated gelatin (G-AA), thiolated gelatin (G-SH), and ODex. A thiol-ene click reaction ensured precursor stability for sufficient tissue penetration, while subsequent crosslinking established a polymer-interlocked structure, significantly improving long-term wet adhesion and demonstrating substantial potential for corneal transplantation [21]. To enhance wet-state adhesion, Tan's team maintained ASO in a low degree of crosslinking and low-viscosity state upon tissue contact, enabling thorough infiltration along collagen-fiber interstices and simultaneous bond formation on both sides of the interface. For long-term stability, polymer interpenetration/interlocking were employed to transmit stress into deeper layers, yielding greater shear resistance. In addition to enhancing mechanical properties, researchers have introduced various functional groups into the GelMA system, thereby further expanding its potential applications. Qian et al. addressed limitations of DA self-polymerization by synthesizing dopamine methacrylamide (DMA)-GelMA, a hydrogel combining strong adhesion, and reactive oxygen species (ROS)-scavenging capabilities to mitigate oxidative stress during corneal regeneration (Fig. 4F) [99].

In ocular surface reconstruction, stem cell-laden biomimetic substitutes are increasingly explored. Huang et al. engineered a GelMA-based corneal stromal analog seeded with human amniotic epithelial stem cell-derived fibroblasts, achieving scar-free healing via microenvironment modulation, proteoglycan secretion, and anti-inflammatory signaling [100]. Nanomaterial integration further optimizes GelMA-based systems. Li et al. incorporated Pluronic F127 diacrylate and Aldehyded Pluronic F127 nanomicelles into collagen-GelMA hydrogels, enhancing adhesion and transparency while addressing weaknesses like low compressive resistance (Fig. 4G) [101].

Alternative chemical modifications beyond methacryloylation are also investigated. Li et al. developed an injectable UV-curable gelatin hydrogel via thiol-acrylate crosslinking, exhibiting higher hydration, biodegradability, and bioactivity than polyethylene glycol (PEG)-, HA-, or alginate-based systems [102]. Sharifi et al. synthesized GELGYM, a glycidyl methacrylate (GMA)-grafted gelatin hydrogel, with biomimetic mechanical and adhesive properties for ocular surface restoration (Fig. 4E) [103].

Gelatin-based materials, such as GelMA, exhibit excellent cell adhesion properties, biocompatibility, and easy processability, making them useful for ocular surface repair. They offer good wet adhesion and are transparent, with potential for in-situ injection. Within this class, the visible-light–curable GelMA platform exemplified by GelCORE is the most clinically advanced. It crosslinks within minutes under blue/green illumination, thereby avoiding UV exposure. The cured network remains optically clear, shows robust wet adhesion, and retains gelatin-derived cell-adhesive motifs that support re-epithelialization [93]. Even so, low mechanical strength and susceptibility to degradation in high-humidity environments challenge long-term stability. Chemical crosslinking, typically used to improve strength, may introduce cytotoxic residuals that affect sensitive ocular tissues. Further research should aim at optimizing crosslinking strategies to balance adhesion and mechanical integrity without compromising ocular safety.

#### 4.1.4 Decellularized corneal stroma matrix-based bioactive materials for the ocular surface

Although various bioactive materials have been used to fill corneal defects, their biochemical composition differs from that of natural corneas, and they fail to replicate the precise arrangement of corneal collagen fibers, limiting their ability to fully mimic the effects of corneal transplantation. Allogeneic corneas, while biologically similar, face challenges such as donor scarcity and immune rejection. In contrast, decellularized xenogeneic corneal matrices, particularly those derived from animals, offer a promising alternative by preserving the extracellular matrix (ECM) components essential for tissue regeneration. These matrices retain pro-regenerative and anti-fibrotic factors, which reduce inflammation and scar formation, thereby enhancing corneal stromal reconstruction [104]. Among these, acellularized porcine corneal stroma (APCS) is the most widely used substitute due to its abundant availability and structural similarity to human corneas in terms of thickness and collagen organization.

The preparation of decellularized corneal matrices involves tissue isolation, cell removal, repopulation with healthy cells, and transplantation. Common decellularization methods include physical (e.g., freeze-thaw cycling [105], electrophoresis [106], high hydrostatic pressure [107], and ultrasonication [108]), chemical [109] (e.g., acetic acid, formic acid, ethanol, and ethylene diamine tetraacetic acid [EDTA]), and enzymatic [110] (e.g., trypsin and phospholipase A2 [PLA2]) approaches. While physical methods minimally disrupt ECM architecture, they often leave residual cellular debris. Chemical agents, however, may degrade ECM components such as collagen and glycosaminoglycans, and residual solvents can induce cytotoxicity or immune reactions. Enzymatic decellularization requires precise control to avoid excessive ECM damage, typically followed by nuclease treatment to eliminate residual DNA.

Despite their advantages, decellularized corneal matrices still require suturing during transplantation and may not conform perfectly to irregular defects due to their rigid structure. To address these limitations, decellularized corneal matrix hydrogels have emerged as a superior alternative. These hydrogels, composed primarily of collagen, can be injected directly into corneal defects, ensuring uniform coverage and strong tissue adhesion. Additionally, they allow for scalable production, functional modification (e.g., crosslinking for enhanced strength), and incorporation of bioactive molecules, offering significant advantages over conventional decellularized grafts.

**Fig. 5:**
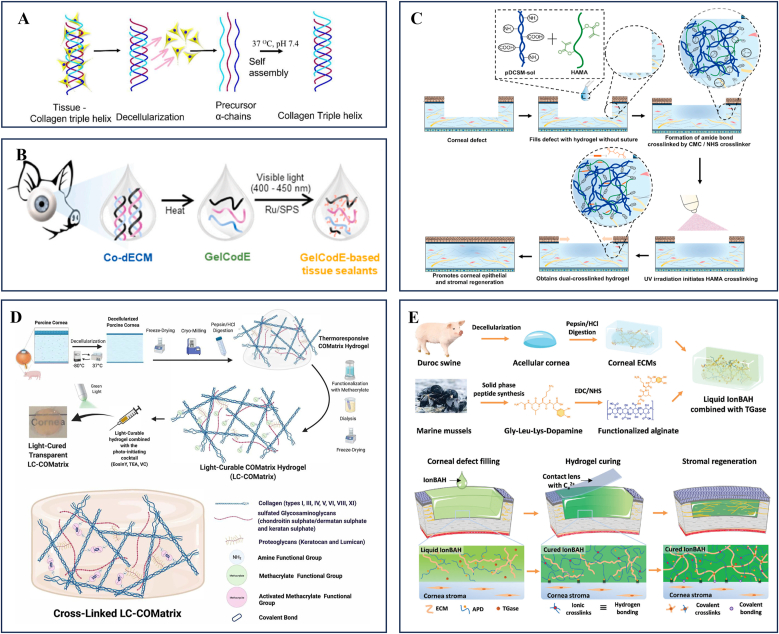
Decellularized corneal stroma matrix-based bioactive materials for the ocular surface A Schematic representation of the sample processing for decellularization. Reprinted with permission from Ref. [111]. B Schematic illustrations showing the gelatinized corneal decellularized extracellular matrix (GelCodE)-based tissue sealants. Reprinted with permission from Ref. [109]. C Schematic illustrations of the hybrid hydrogel applied onto corneal defect in situ for long-term regenerative repair. Reprinted with permission from Ref. [20]. D The fabrication process of Light-Curable COMatrix (LC-COMatrix) Hydrogel. Reprinted with permission from Ref. [112]. E Schematic illustration and ultrastructure of IonBAH. The corneal ECMs are separated from fresh Duroc porcine corneas by protective decellularization and enzymatic digestion treatment. Inspired by mussel adhesion protein, tetraceptides (Gly-Leu-Lys-Dopamine) are artificially synthesized and grafted onto the alginate backbone. The liquid IonBAH is composed of corneal ECMs, functionalized alginates (APD), and transglutaminases (TGases). Reprinted with permission from Ref. [113].

Recent studies demonstrate that APCS-derived hydrogels exhibit excellent bio-safety, adhesion, and regenerative potential, making them ideal for corneal repair. Zhou et al. developed a self-gelling hydrogel via pH and salt concentration modulation, which promoted short-term wound healing in mice but lacked sufficient mechanical strength for large defects [110]. Chameettachal et al. fabricated a human decellularized corneal hydrogel that accelerated epithelial regeneration, though it shared similar mechanical limitations (Fig. 5A) [111]. Yazdanpanah et al. enhanced hydrogel stability by functionalizing decellularized porcine corneal matrix with methacrylic anhydride (LC-COMatrix), enabling visible light crosslinking without compromising transparency or bioactivity (Fig. 5D) [112]. Shen et al. further enhanced the mechanical properties of the hydrogel by introducing a dual photo-chemical crosslinking system composed of N-cyclohexyl-N’-(2-morpholinoethyl) carbodiimide metho-p-toluenesulfonate/N-hydroxysuccinimide (CMC/NHS) for chemical crosslinking, along with phenyl-2,4,6-trimethylbenzoylphosphinate (LAP) for photo-crosslinking, resulting in a denser network structure with superior tissue adhesion (Fig. 5C) [20]. Kim et al. employed thermal denaturation and Ru/SPS-mediated visible light crosslinking to develop gelatinized cornea-derived extracellular matrix (GelCodE), which maintained corneal transparency while enabling scar-free regeneration (Fig. 5B) [109]. Zhao et al. combined decellularized corneal solution with functionalized alginate (APD) and transglutaminase (TGase), creating a dual ionic-covalent network that avoided toxic crosslinkers and enhanced wet adhesion, proving effective for large (6 mm) defects in long-term animal studies (Fig. 5E) [113].

Although existing technologies such as 3D printing and electrospinning have partially replicated the corneal architecture, reproducing its intricate lamellar ultrastructure with high precision remains a significant challenge. In response to this limitation, Zhao et al. developed a humanized corneal stroma-like adhesive patch (HCSP) [114]. Their strategy began with the use of APCS as a structural template, from which they fabricated a nanotubular hydrogel skeleton primarily composed of polyethylene glycol diacrylate (PEGDA) via a multistep process. This scaffold was then integrated with a human-derived corneal extracellular matrix (hCECM) and further functionalized with GelMA to achieve strong interfacial adhesion. The resulting HCSP successfully replicated the ordered lamellar alignment and optical transparency of native human corneal stroma. In addition, it exhibited significantly improved performance compared to conventional hydrogels. Specifically, HCSP was more effective in maintaining the phenotypes of both epithelial and stromal cells, enhancing epithelial regeneration, and suppressing fibrotic transformation.

Despite their adhesive and modifiable properties, decellularized corneal hydrogels suffer from irreversible disruption of native collagen alignment, resulting in reduced mechanical strength and loss of anisotropic optical properties. Future research should focus on structural biomimicry and bioactive modulation (e.g., incorporating stem cells, growth factors, and anti-scarring agents) to optimize their clinical applicability.

Decellularized corneal stroma matrix-based materials are highly advantageous for their native ECM-like structure, which promotes corneal regeneration and reduces scarring. They maintain pro-regenerative and anti-fibrotic properties, offering potential for large-scale repair. However, they still require suturing during transplantation and may not adapt perfectly to irregular defects due to their rigid structure. Recent advances in decellularized corneal hydrogels have improved uniform coverage and mechanical strength, but standardization and the control of degradation rates remain a challenge. Further refinement in their biomechanical properties and functional modification is necessary.

### 4.2 Polysaccharide-based bioactive materials for the ocular surface

Polysaccharide-based bioactive materials exhibit low toxicity and high biocompatibility, as they degrade in vivo without producing toxic byproducts. Certain polysaccharides also possess additional therapeutic properties, including antioxidant, immunomodulatory, hemostatic, and anti-inflammatory effects, making them particularly suitable for the development of ocular adhesive bioactive materials [[115], [116], [117], [118]].

#### 4.2.1 HA-based bioactive materials for the ocular surface

HA is a naturally occurring polysaccharide ubiquitously present in human tissues [119]. As an essential component of the ECM, HA exhibits excellent biodegradability and gel-forming properties. Beyond its anti-inflammatory and antioxidant effects [120], HA facilitates cellular signaling, promotes ECM synthesis, and accelerates re-epithelialization and wound healing [121]. These attributes make HA a promising candidate for bioadhesive applications. However, a key limitation of conventional HA-based hydrogels is their rapid clearance from the ocular surface, resulting in transient therapeutic efficacy. To address this, photo- or chemical crosslinking strategies have been explored to enhance hydrogel stability and prolong retention time, representing a major research focus in HA-based hydrogel development.

Thiolated carboxymethyl hyaluronic acid (CMHA-S) can be crosslinked via thiol-reactive agents or disulfide bond formation to produce hydrogels, films, or sponges, demonstrating favorable outcomes in animal models of corneal epithelial abrasion and alkali burns [122,123]. Lee et al. utilized blue light in the presence of riboflavin phosphate (RFP) to initiate a thiol-ene reaction between methacrylated hyaluronic acid (MA-HA) and thiolated hyaluronic acid (SH-HA), forming an in situ HA hydrogel with delayed gelation. This system retained fluidity post-irradiation before gradually solidifying within minutes, offering clinical advantages by minimizing direct light exposure to tissues [40].

To further improve mechanical strength, Yang's team incorporated methacrylate (MA) and 4-pentenoate (PA) as photocrosslinkable groups into HA chains [22]. Short-chain MA provided high crosslinking density and rigidity, while long-chain PA enhanced flexibility and elongation, reducing brittleness and preventing rapid fracture under stress. This dual-crosslinking design enabled rapid gelation followed by progressive reinforcement. Wang et al. adopted a temperature-triggered primary gelation followed by visible light secondary curing [38]. Inspired by mussel-inspired wet adhesion, their hydrogel, composed of F127-diacrylate (F127-DA) and DOPA-modified methacrylated HA (HAMA-DOPA), initially formed a physical gel upon corneal application, rapidly sealing wounds before light-induced radical crosslinking stabilized the structure without compromising adhesion in aqueous environments. To mitigate DOPA-induced discoloration from oxidation, the authors optimized rapid crosslinking, pH modulation, and low-density DOPA grafting, ensuring optical transparency for ophthalmic use. Fernandes-Cunha et al. employed a non-covalent supramolecular strategy using cyclodextrin- and adamantane-grafted HA, achieving reversible dynamic crosslinking via host-guest interactions [23]. This shear-thinning hydrogel demonstrated cell-friendly nature, facilitating injection and corneal repair. For emergency scenarios (e.g., battlefield injuries), Kambhampati et al. developed OcuPair, an in situ photocurable adhesive hydrogel comprising methacrylated hydroxyl polyamidoamine (PAMAM) dendrimers and HA [124,125]. It forms a transparent, flexible, and robust corneal bandage within 90 s, with superior strength, intraocular pressure resistance, and ease of application/removal compared to traditional fibrin glue or cyanoacrylate adhesives.

HA-based bioactive materials offer excellent biodegradability and cell signaling properties. They are highly biocompatible, anti-inflammatory, and promote tissue healing. However, native HA degrades too rapidly on the ocular surface, which limits its long-term therapeutic effects. Recent advancements in photo- or chemical crosslinking have significantly enhanced the stability and retention time of HA-based hydrogels. Still, issues related to crosslinking safety and the selection of initiators must be carefully considered. Additionally, prolonged retention in the ocular surface remains a concern.

#### 4.2.2 Dextran-based bioactive materials for the ocular surface

Dextran, a polysaccharide characterized by high biocompatibility, biodegradability, and low immunogenicity, can be readily modified with various polymers and therapeutic agents due to its diverse functional groups. For instance, ODex contains abundant aldehyde groups, enabling dynamic chemical linkages with tissues via Schiff base reactions with amino (or hydrazine/hydrazide) groups under mild conditions [21]. Dextran facilitates tissue repair through multiple mechanisms, serving as an artificial niche for cell culture and differentiation, a scaffold for cell delivery, and a modulator of collagen deposition and tissue remodeling [126].

Numerous materials, including PEG-amine, collagen, gelatin, ε-poly(L-lysine) and amine-terminated PAMAM dendrimers, can provide amino groups for dextran-based bioactive materials. Among these, materials derived from collagen, gelatin, and PEG are discussed separately in their respective sections. Takaoka et al. designed a chemically defined bioadhesive for sutureless keratoplasty, composed of ε-poly(L-lysine) and aldehyde-functionalized dextran, forming a transparent and flexible hydrogel within 19 s while exhibiting inherent antibacterial properties [127]. To overcome the rapid degradation and weak structural integrity of conventional hydrogels, Zhang et al. introduced a triple-crosslinked hydrogel (T-AlgDD) for corneal adhesives, combining methacrylated alginate (AlgMA), aldehyde-functionalized dextran (DexCHO), PAMAM dendrimers, and Ca^2+^. This system integrates photoinitiated radical polymerization, Ca^2+^-mediated ionic crosslinking, and Schiff base reactions, yielding a material with superior mechanical toughness and tunable adhesion [128].

Dextran-based bioactive materials benefit from high biocompatibility and easy modification with other polymers. Their Schiff base reactions allow for dynamic tissue bonding. Multi-mechanism synergy is often required to stabilize the interface, making complex formulations and processing techniques necessary. Long-term stability and easy application are areas that need further improvement.

#### 4.2.3 Chitosan-based bioactive materials for the ocular surface

Chitosan (CS), a cationic linear polysaccharide derived from chitin deacetylation, consists of β-(1  →  4)-linked D-glucosamine and N-acetyl-D-glucosamine units. This naturally occurring biopolymer exhibits exceptional biocompatibility, minimal immunogenicity, and controlled biodegradability, making it particularly valuable for ophthalmic applications. Its inherent antimicrobial properties and capacity to enhance drug permeation have positioned CS as a promising candidate for ocular drug delivery systems [129,130].

The presence of reactive hydroxyl and amino groups in CS facilitates chemical modifications to optimize its properties for ocular surface applications. Wang et al. demonstrated enhanced hydrophilicity and cell adhesion through carbodiimide-mediated conjugation of HA to CS [131]. Similarly, Li et al. developed collagen-CS composite films via carbodiimide crosslinking, combining the mechanical strength of CS with collagen's hydrophilic characteristics, showing potential as corneal tissue engineering scaffolds [132]. Addressing toxicity concerns associated with conventional crosslinkers, Shah et al. employed natural dual crosslinking agents (tannic acid and genipin) to create DC-Col/Chi films suitable for ocular surface reconstruction [133].

For corneal wound closure, innovative CS-based adhesives have been developed. Tan et al. designed a laser-activated CS film using indocyanine green (ICG) as a photosensitizer, achieving secure corneal incision sealing [134,135]. Lee et al. alternatively utilized methylene blue-activated kynurenine-modified CS adhesives that promoted wound healing while maintaining optical clarity [136]. Beyond wound closure, CS-based membranes show therapeutic potential for chemical burns. Ye et al. fabricated electrospun nanofibrous membranes surface-modified with CS, which enhanced corneal epithelial regeneration while reducing scar formation [137].

The optical transparency and biodegradability of CS make it ideal for corneal tissue engineering scaffolds. Tayebi et al. incorporated chitosan nanoparticles (CSNPs) into CS/polycaprolactone (PCL) composite films, creating scaffolds with human decellularized corneal matrix-like transparency that maintained corneal endothelial cell phenotype [138]. For stromal defects, Tang et al. developed CS hydrogels loaded with induced pluripotent stem cell-derived mesenchymal stem cell (iPSC-MSC) exosomes [139]. The sustained release of exosomal miR-432-5p targets the TRAM2 signaling pathway, regulating collagen biosynthesis and suppressing the fibrotic cascade in corneal stromal stem cells (CSSCs). This mechanism effectively inhibits abnormal ECM deposition, thereby mitigating scar formation. Similarly, Wei et al. engineered dynamically crosslinked hydrogels using oxidized guar gum and carboxymethyl CS, which demonstrated superior corneal healing through controlled release of mesenchymal stem cell-derived exosomes that simultaneously attenuated scar formation and inflammation [140].

Chitosan-based materials are known for their antimicrobial properties, biocompatibility, and ease of chemical modification. They are capable of promoting epithelialization and tissue regeneration. However, their low mechanical strength and pH/ionic sensitivity are limiting factors. Crosslinking agents, which are used to enhance adhesive strength, may cause toxicity issues in certain formulations. Future work should focus on biocompatible crosslinking agents and improving the mechanical performance of these materials.

### 4.3 Synthetic bioactive materials for the ocular surface

Synthetic bioactive materials for the ocular surface primarily comprise cyanoacrylate tissue adhesives (CTAs) and PEG-based bioactive materials. Due to suboptimal acceptance by the host organism, CTAs are no longer the preferred choice for ocular surface applications. In contrast, PEG-based materials have emerged as a focal point in research, with ongoing modifications to enhance their adaptability to increasingly complex ocular surface requirements.

#### 4.3.1 Cyanoacrylate-based bioactive materials for the ocular surface

CTAs are primarily composed of alkyl cyanoacrylate monomers, which are esters of α-cyanoacrylic acid synthesized through the condensation of cyanoacetic acid esters and formaldehyde [12]. In ophthalmic surgery, CTAs serve as alternatives to sutures and are applied in various contexts, such as cataract incision repair [141], retinal detachment management [142], scleral reinforcement [143], extraocular muscle attachment [144], and corneal perforation closure [145]. Notably, despite being an off-label use, CTA remains one of the principal therapeutic options for corneal perforation. As a synthetic bioactive adhesive material for ocular applications, CTA exhibits one of the highest mechanical strengths among its class [77], a characteristic well-supported by extensive clinical studies [8,9,14,15,[145], [146], [147]].

A key advantage of cyanoacrylates lies in their rapid application, which involves hydroxyl-initiated polymerization to form a strong film that effectively seals wounds and maintains globe integrity [148]. However, poor compatibility with living tissues remains the primary barrier to their widespread clinical use. Upon in situ polymerization, CTAs form impermeable, solid masses that may provoke local irritation and inflammation, potentially leading to complications such as giant papillary conjunctivitis and corneal neovascularization. The highly exothermic curing process generates opaque, rough masses with poor corneal integration and inadequate permeability, impairing wound healing and visual recovery. Moreover, the degradation of CTAs releases toxic by-products, notably cyanoacetic acid esters and formaldehyde, whose accumulation in tissues contributes to significant cytotoxicity [149]. To address this, researchers have developed derivatives such as butyl-, heptyl-, methoxypropyl-, and octyl-cyanoacrylates, which degrade more slowly and exhibit reduced tissue toxicity due to their longer alkyl chains [150].

It is important to note that CTA applications are typically temporary, with most patients eventually requiring secondary surgical intervention. For example, the median retention time for CTA on the corneal surface in the treatment of perforation or thinning is approximately 29 days [11]. Over time, therapeutic efficacy diminishes: 72 % of treated eyes remain intact without surgical intervention at 10 days post-application, but this rate drops to 46 % by day 90. Given the potential toxicity, manual removal is recommended if spontaneous detachment of CTA does not occur following treatment [148].

Cyanoacrylate-based bioactive materials offer superior adhesion strength and fast application, making them suitable for emergency scenarios such as corneal perforation. However, their poor tissue compatibility and long-term toxicity limit their use, particularly due to heat release and foreign-body reactions. Moreover, their rigid nature can lead to discrepancies between the adhesive and the ocular tissue, resulting in tissue damage or retinal injury in the fundus. The material's limited durability also necessitates secondary treatments, and further refinement in biocompatibility and degradation control is crucial.

#### 4.3.2 PEG-based bioactive materials for the ocular surface

PEG is a non-toxic, hypoallergenic polymer widely used in ophthalmic surgery and wound repair due to its advantageous properties [151]. First, PEG contains abundant hydroxyl groups, conferring excellent hydrophilicity that minimizes ocular irritation while facilitating the permeation of small molecules [152,153]. Its chemical modifiability further enhances its versatility. Additionally, PEG's high optical transparency ensures unimpaired postoperative visual recovery. Importantly, PEG undergoes programmed degradation in vivo, eliminating long-term ocular retention and reducing the risk of foreign body reactions [154]. However, PEG-based sealants often require multi-component mixing and exhibit a short application window, complicating clinical use. For instance, ReSure®, a dual-component system comprising PEG and trilysine acetate, must be applied within 30 s of polymerization initiation [155].

ReSure® is the most widely used PEG-based bioactive material, composed of a four-arm PEG-NHS prepolymer and a trilysine acetate crosslinker. Upon mixing, it forms a stable hydrogel within 30 s. This material is hydrophilic, non-toxic, minimally immunogenic, and chemically modifiable, naturally sloughing off with tear film during epithelialization without requiring manual removal. Approved by the FDA for sealing clear corneal incisions ≤3.5 mm, ReSure® has demonstrated efficacy comparable or superior to sutures in preventing ocular fluid leakage across diverse procedures, including Descemet's stripping endothelial keratoplasty (DSEK) [155], pterygium surgery [156], Descemet's membrane endothelial keratoplasty (DMEK) [157], intrastromal corneal ring segment (ICRS) implantation [158], trabeculectomy [159], and phacoemulsification with or without glaucoma surgery [[160], [161], [162]]. Despite its advantages, ReSure®’s sealing effect lasts only 1–3 days, limiting its utility to short-term, small-incision applications.

PEG can be combined with other components to form composite hydrogels with enhanced properties. For example, Bhatia et al. developed a dextran-based tissue adhesive primarily for corneal incision sealing, where aldehyde groups from ODex react with PEG-amine to achieve rapid in situ crosslinking under physiological conditions, offering strong adhesion, low cytotoxicity, controlled degradability, and ease of application [163,164]. Strehin et al. modified chondroitin sulfate into chondroitin sulfate-NHS, which covalently crosslinks with PEG-(NH)_6_ to form a gel-like adhesive (chondroitin sulfate-PEG), effectively sealing corneal incisions without significant adverse effects [165]. Building on this, Chae et al. incorporated a collagen vitrigel (CV) into the chondroitin sulfate-PEG adhesive, creating a composite system capable of sealing larger wounds (6–8 mm), showing promise for battlefield penetrating eye injuries [166]. Beyond symmetric PEG composites aimed mainly at incision sealing, Qie et al. reported an asymmetric Janus PEG-based hydrogel tailored for suture-free corneal defect repair [167]. The bottom PPH layer (four-arm PEG-NH_2_/PEG-succinimidyl carbonate crosslinked with oxidized heparin) electrostatically scavenges pro-inflammatory and pro-fibrotic cytokines to downregulate the wound-healing cascade and suppress scarring, while the spray-coated, UV-crosslinked thiolated ε-poly-L-lysine (EPL-SH) top layer provides broad-spectrum antibacterial protection against MRSA and *P. aeruginosa*. Interfacial covalent coupling (Schiff base and amide formation) plus a partial IPN ensures robust wet adhesion and burst pressures exceeding intraocular pressure, enabling stable, transparent coverage and long-term anti-fibrosis/anti-infection benefits in vivo.

**Fig. 6:**
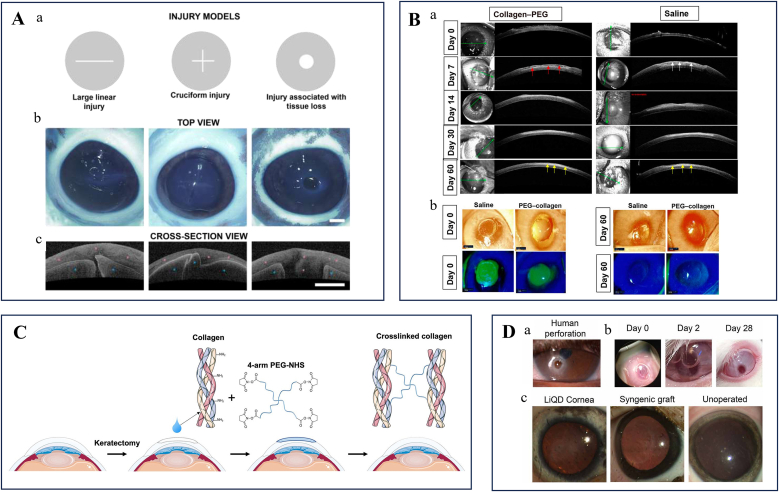
Polyethylene glycol-based bioactive materials for the ocular surface. A Application and dimensional assessment of hydrogel patches on different types of open globe injuries. (a) Models of open-globe injury models tested, (b) Macroscopic images and (c) OCT images of pig eyeballs with different injury models after application and photocrosslinking for 4 min (n = 3 per group). Blue asterisks correspond to corneal tissue and pink asterisks to the hydrogel patch. Scale bar = 3 mm. Reprinted with permission from Ref. [174]. B Evaluation of corneal wound healing at day 60. a, Saline-treated and collagen–PEG-treated corneas were analyzed using OCT. Red arrows show the collagen–PEG gel on day 7. White arrows indicate epithelial hyperplasia in the saline group at day 7. Large yellow arrows at day 60 show the regenerated epithelial layer, proliferated cells, and scars. b, Evaluation of the cornea under bright light before keratectomy, after applying the collagen–PEG gel, and at day 60 after surgery. The wound area was visualized using fluorescein at days 0 and 60. Reprinted with permission from Ref. [172]. C Schematic diagram showing the injection of the in situ-forming polyethylene glycol-collagen hydrogel into an in vivo rabbit corneal defect model. Reprinted with permission from Ref. [171]. D (a) Example of a human corneal perforation. (b) Postsurgical photos of rabbits immediately after injecting LiQD Cornea into a perforated cornea. The two-stepped surgically induced perforation can be seen. At day 2 after surgery, the air bubble placed under the cornea during surgery is prominent, indicating that the perforation was completely sealed. The perforated cornea was completed healed by 28 days after operation. Photo credit: Damien Hunter, University of Sydney. (c) Mini-pig corneas where the LiQD Cornea was tested as an alternative to a donor allograft, showing the gross appearance of the LiQD Cornea, syngeneic graft, and an unoperated eye at 12 months after surgery. Reprinted with permission from Ref. [168].

Collagen is another common PEG composite partner, as PEG crosslinking improves collagen's stability, degradation profile, and tunable physicochemical properties. Samarawickrama et al. synthesized a collagen-like peptide (CLP) that reacts with maleimide-functionalized PEG to form CLP-PEG hydrogels. Further crosslinking with 4-(4,6-dimethoxy-1,3,5-triazin-2-yl)-4-methylmorpholinium chloride yielded LiQD Cornea, a biocompatible hydrogel supporting wound closure, stromal regeneration, and corneal nerve repair, offering a potential alternative to lamellar keratoplasty (Fig. 6D) [[168], [169], [170]]. Similarly, Chun et al. developed a PEG-collagen hydrogel via amide bonding between multi-arm PEG-NHS and bovine type I collagen, which adapts to irregular corneal defects, promotes epithelialization, and reduces scarring (Fig. 6B and C) [36,171,172].

GelMA-PEG hybrids exhibit superior mechanical strength. Kong et al. infused GelMA into a PCL-PEG copolymer scaffold, creating a 3D hydrogel with optimal mechanical and optical properties while inducing stromal regeneration [173]. Jumelle et al. engineered a hydrogel patch from PEGDA, GelMA, and HAGM, with tunable physical properties via polymer concentration adjustments. This patch outperformed ReSure® in burst pressure, transparency, and degradation duration, making it suitable for diverse ocular injuries (Fig. 6A) [174].

PEG-based materials also serve as stem cell carriers for treating limbal stem cell deficiency and corneal endothelial dysfunction. Li et al. incorporated PEG into SF to enhance membrane roughness, tensile strength, and cell adhesion. Limbal epithelial stem cells (LESCs) cultured on PEG-modified SF membranes successfully reconstructed the corneal limbal niche in rabbits, enabling functional recovery and secondary repair post-injury [175]. Ozcelik et al. developed PEG-based hydrogel films (PHFs) crosslinked via sebacoyl chloride esterification, reinforced with PCL for mechanical stability. PHFs adhered well to corneal endothelia without significant inflammation, showing promise for endothelial cell transplantation [176]. Further functionalization has led to the development of smart hydrogel. Tsurkan et al. designed a starPEG-chondroitin sulfate hydrogel with a factor Xa-cleavable peptide sequence, allowing gentle cell detachment without disrupting cell-matrix junctions [177].

PEG-based materials offer flexibility, optical transparency, and biocompatibility. Their ability to be chemically modified makes them versatile for ocular applications, particularly for short-term sealing and drug delivery. However, multi-component mixing and short application windows complicate their clinical use. Long-term adhesion, especially in wet environments under shear forces, remains a challenge. Additionally, their durability is limited by weak long-term mechanical properties, and more stable formulations are needed for effective clinical use.

## 5 Adhesive bioactive materials for the ocular fundus

RRD is a vision-threatening condition and the most prevalent form of retinal detachment [178]. This pathology primarily results from retinal break formation, allowing subretinal fluid accumulation and subsequent separation of the neurosensory retina from the underlying retinal pigment epithelium (RPE). Standard therapeutic interventions include SB, PPV, and pneumatic retinopexy. While these conventional approaches demonstrate high success rates in uncomplicated cases, intraocular tamponade agents (e.g., gases or silicone oil) exhibit suboptimal efficacy in treating inferior retinal breaks and may induce complications such as band keratopathy, glaucoma, cataract, light scattering, or vascular occlusion [[179], [180], [181], [182], [183]]. Moreover, postoperative positioning requirements for gas or silicone oil tamponade pose significant challenges for elderly patients or those with spinal disorders. These limitations have spurred investigations into alternative strategies. Notably, adhesive bioactive materials for the ocular fundus represent a promising advancement, enabling localized sealing of retinal breaks without tamponade dependency. This approach eliminates postoperative positioning constraints, mitigates complication risks, and enhances patient comfort while maintaining effective retinal reattachment outcomes (Table 3).

**Table 3:** Characteristics and evidence overview of ocular fundus adhesive bioactive materials.

Material Category	Typical Indications	Delivery/Handling Pathway	Curing/Triggering Method	Observed Adhesion Time	Adjunctive Therapy	Main Advantages	Main Limitations	Evidence Level
Fibrin-based	Retinal tears; MD	Post-vitrectomy filling/drops	Two-component coagulation	4–6 days; some reports up to months	Laser/gas	Biocompatible; independent of tissue health	Short adhesion duration, weak mechanical strength, rapid degradation	Multicenter clinical trials
Platelet-based	Refractory MH	Post-vitrectomy or drops	Platelet aggregation	/	Gas tamponade	Accelerates healing, enhances tissue repair	No significant short-term visual improvement; secondary outcomes limited	Randomized trials
Gelatin-based	MH; intravitreal plugs	Intravitreal injection	Enzyme/UV/chemical	Weeks-months (up to 6 months)	Gas/laser	Good biological compatibility and processing	Long-term mechanical stability still needs improvement	Animal/early clinical
HA-based	Retinal tear; vitreous substitute	Minimally invasive needle injection	Chemical crosslinking	2–3 week; lasts months in some cases	Laser	Easy injection, transparent, long-term potential for drug release	Some formulations have shown IOP fluctuation	Animal/clinical case studies
PEG-based	Retinal tear sealing; gas substitute	Intravitreal or via sclera injection	NHS-PEG chemistry or light-curing	2–4 weeks	Laser/gas	Instant closure, light-curing	Inflammation/PVR risks	Animal/limited clinical
Cyanoacrylate	Persistent retinal tears; external SB reinforcement	Intravitreal injection	In-situ polymerization on wet surface	2–90 days	/	High bonding strength, fast	Risk of retinal damage, heat release, mismatch in tissue biomechanical properties	Animal/early clinical

**Abbreviation**s: RRD: rhegmatogenous retinal detachment, MD: macular detachment, MH: macular hole, UV: ultraviolet, HA: hyaluronic acid, IOP: intraocular pressure, PEG: polyethylene glycol, NHS: N-hydroxysuccinimide, PVR: proliferative vitreoretinopathy, SB: Scleral buckling.

Unlike the semi-wet, mechanically dynamic ocular surface, the ocular fundus is a fully hydrated, relatively static environment that requires adhesive materials capable of performing in a wet environment and adhering securely without the complications of shear forces. Adhesives for the fundus must offer extended stability (from weeks to months) and have low heat generation and minimal toxicity, especially when used in conjunction with retinal laser treatments or gas tamponade. The material's degradation profile must align with the healing kinetics of the retina. Materials used in the fundus should ideally allow for sustained drug delivery, vascular reinforcement, or retinal repair without compromising visual function or electrophysiological integrity. Furthermore, these materials should be compatible with the micro-environmental conditions and anatomical constraints of the eye, ensuring long-term retinal stability.

### 5.1 Blood-derived bioactive materials for the ocular fundus

#### 5.1.1 Fibrin-based bioactive materials for the ocular fundus

Fibrin glue has been clinically utilized as a bioactive adhesive for retinal tear closure for three decades [184]. This biomaterial exhibits favorable characteristics including non-toxicity and biodegradability [185,186]. Unlike laser photocoagulation, its efficacy is independent of retinal tissue health status, making it particularly advantageous in cases where conventional laser treatment is contraindicated. Notably, fibrin glue demonstrates superior outcomes compared to traditional tamponade techniques for inferior retinal tears, which are often challenging to manage [187].

Despite these advantages, the clinical utility of fibrin glue is constrained by several limitations. Its relatively short adhesive duration (4–6 days), coupled with suboptimal mechanical strength and rapid degradation kinetics, restricts its application primarily to an auxiliary function in retinal stabilization rather than as an independent fixation modality. Clinical evidence from Tyagi et al. supports its efficacy when combined with laser photocoagulation as an alternative to silicone oil or gas tamponade for rhegmatogenous RRD, with reported success in achieving retinal reattachment [187]. These findings are corroborated by Wang et al.'s clinical investigations [188]. Interestingly, Aydin et al. documented a unique case where fibrin glue alone, without supplemental laser treatment or tamponade, successfully maintained retinal reattachment for two years postoperatively in an RRD patient [189]. Furthermore, preliminary studies have extended its application to optic disc pit-associated macular detachment, showing promising results [190].

#### 5.1.2 Platelet-based bioactive materials for the ocular fundus

MH, a full-thickness defect in the neurosensory retina at the foveal center, typically manifests as varying degrees of visual acuity loss and metamorphopsia [[191], [192], [193]]. Despite advancements in surgical techniques, the treatment success rate for refractory and recurrent MHs remains suboptimal. A key contributing factor is that MH repair depends not only on anatomical closure but also on cellular proliferation, particularly involving Müller cells and astrocytes [194,195].

**Fig. 7:**
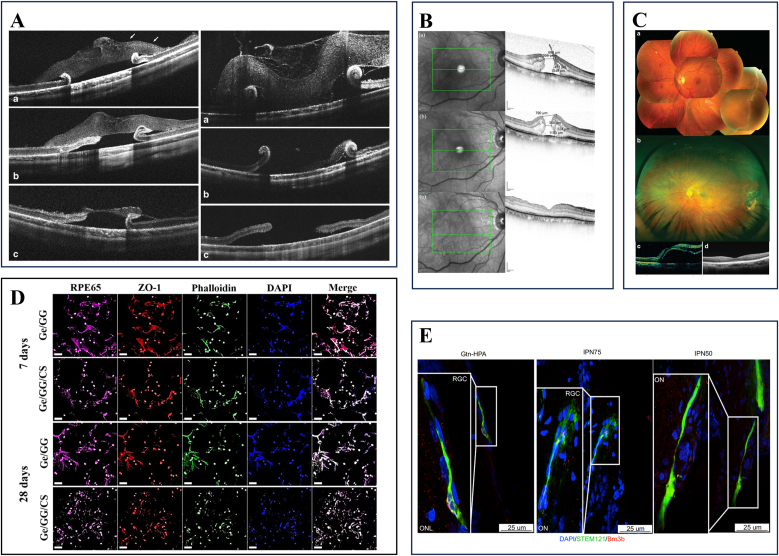
Adhesive bioactive materials for the ocular fundus. A Optical coherence tomography (OCT) findings following vitrectomy with application of a gelatin–mTG complex (left) or gelatin alone (right): (a) One day postoperatively; (b) three days postoperatively; (c) seven days postoperatively. Reprinted with permission from Ref. [213]. B Anatomical closure of a persistent macular hole with autologous platelet concentrate and sulfur hexafluoride 20 % gas tamponade. (a) the idiopathic macular hole before surgery; (b) the persistent macular hole before surgery; (c) the result after re-vitrectomy with autologous platelet concentrate (APC) and sulfur hexafluoride 20 % gas tamponade. Reprinted with permission from Ref. [206]. C Fundus photographs and optical coherence tomography images from a case that cover retinal break Seprafilm. Fundus photographs of the left eye preoperatively (a) and 9 years postoperatively (b). Horizontal OCT images of the left eye through the macula preoperatively (c) and 9 years postoperatively (d) Reprinted with permission from Ref. [232]. D Immunofluorescence staining of RPE65, ZO-1, phalloidin, and DAPI on 7 and 28 days of cells cultured in Ge/GG/CS hydrogel. Reprinted with permission from Ref. [214]. E Fluorescence microscopy images of retina sections 1 month after transplantation using Gtn-HPA hydrogel as a cell delivery vehicle. Slides were stained with DAPI (nuclei staining), STEM121 (human marker), and Brn3b (RGC marker). Immunohistochemistry shows cell bodies expressing human marker extending processes inside the retina towards the optic nerve, suggesting engraftment, for all samples injected in hydrogels. Reprinted with permission from Ref. [215].

Autologous platelet concentrate (APC), obtained through centrifugation of the patient's blood, facilitates tissue repair via two primary mechanisms: (1) platelet aggregation forms a biological scaffold at the surgical site, aiding mechanical wound closure [196], and (2) the release of growth factors such as platelet-derived growth factor (PDGF), angiogenic factors, and transforming growth factor-β (TGF-β) [197,198], which stimulate cellular proliferation, ECM remodeling, and neovascularization. Given its intrinsic bioactive properties, APC, a naturally derived substance, has been explored as a supplementary therapeutic strategy in MH surgery (Fig. 7B).

Clinical studies evaluating APC's safety and efficacy have demonstrated significantly higher anatomical closure rates in the APC-augmented surgery group compared to conventional surgery alone, with no significant difference in complication rates [[199], [200], [201], [202]]. However, no statistically significant differences were observed in secondary outcomes, including six-month recurrence rates, visual acuity, or visual field loss [[203], [204], [205], [206], [207], [208], [209]]. These findings suggest that while APC may improve anatomical outcomes, its benefits do not necessarily translate into substantial short-term visual improvement, unlike other established MH treatments.

### 5.2 Gelatin-based bioactive materials for the ocular fundus

Gelatin-based bioactive materials have demonstrated significant potential in retinal repair, particularly for reattaching detached retinas and sealing MHs. Peyman et al. developed a partially cross-linked gelatin-derived absorbable macular plug, which proved highly effective in patients with high myopia complicated by posterior staphyloma, achieving a 100 % anatomical reattachment rate postoperatively [210]. Chen et al. investigated the applicability of GelMA for retinal hole closure, finding that a 75 % substitution degree and 20 % concentration provided optimal adhesion strength, stability, and degradation kinetics without inducing severe intraocular complications, thus positioning it as an ideal candidate for retinal repair [211]. Beyond photocrosslinking, chemically cross-linked gelatin-based materials have also been explored. Yamamoto et al. utilized microbial transglutaminase (mTG) to catalyze amide bond formation between gelatin molecules, producing a gelatin-mTG composite with progressive degradation rates, non-toxic byproducts, and sufficient wet adhesion strength. In a rabbit retinal hole model, this material remained attached for over one week while maintaining retinal apposition, highlighting its potential for vitreoretinal surgery (Fig. 7A) [212,213].

Owing to their favorable interaction with biological systems and low immunogenicity, gelatin-based bioactive materials are widely employed as scaffolds in tissue engineering, providing structural support and a conducive microenvironment for cell growth—a key focus in retinal regenerative medicine. Rim et al. reinforced gelatin networks with gellan gum (GG) and CS, creating a Ge/GG/CS hydrogel that effectively encapsulated and preserved RPE morphology (Fig. 7D) [214]. Similarly, Park et al. covalently grafted hydroxyphenylpropionic acid (HPA) to gelatin, yielding a Gtn-HPA hydrogel for retinal precursor cell (RPC) delivery to the subretinal space (Fig. 7E). This hydrogel exhibited strong adhesion to the retinal inner limiting membrane, high cell retention, minimal immune activation, and enhanced safety [215,216]. Tang et al. further advanced this approach by combining HAMA, thiolated gelatin (Gel-SH), and PDA nanoparticles with RPCs, forming an in situ 3D hydrogel network via Michael addition under physiological conditions. This system not only facilitated RPC delivery but also promoted their differentiation into photoreceptor neurons, improving migration and integration into retinal tissue [217]. Beyond cell delivery, adhesive bioactive materials can also enable sustained drug release in ocular applications. For instance, Liu et al. modified gelatin with cinnamic acid to produce a photocurable hydrogel encapsulating curcumin-loaded PDA nanoparticles (Cur@PDA@GelCA). This injectable formulation remained stable in the subretinal space, leveraging PDA and curcumin to synergistically scavenge ROS, thereby addressing a critical gap in optic nerve crush therapy [218].

### 5.3 HA-based bioactive materials for the ocular fundus

PPV is the standard surgical intervention for treating retinal diseases. However, following the removal of the natural vitreous body, a substitute is required to maintain the structural integrity of the eyeball and ensure retinal attachment. Conventional vitreous substitutes, such as gases (e.g., sulfur hexafluoride) and silicone oil, offer short-to medium-term support but are often associated with complications, including visual impairment, postural limitations, and long-term toxicity. To overcome these challenges, researchers have proposed that an ideal vitreous substitute should exhibit high transparency, a suitable refractive index, biodegradability, and injectability.

HA, owing to its natural hydration properties, biodegradability, and non-toxicity to biological systems, has been considered a potential candidate in ophthalmic applications for nearly four decades [219]. Beyond its use as a vitreous substitute, HA-based bioactive materials have also been developed for retinal tissue repair and as vehicles for cell delivery. Although native HA degrades rapidly in the ocular environment—rendering it unsuitable for extended use—chemical modification through crosslinking techniques, such as aldehyde-hydrazone, hydrazide, photo-crosslinking, and oxime reactions, enhances its structural stability. These modifications enable the formation of low-toxicity, highly viscous, and injectable hydrogels that exhibit excellent performance in maintaining intraocular pressure and preserving retinal architecture [[219], [220], [221], [222], [223], [224], [225]].

A notable example is Healaflow®, a commercially available hydrogel formed by etherification between HA and 1,4-butanediol diglycidyl ether (BDDE). Its high water content and refractive index closely mimic the optical and physical properties of the natural vitreous, enabling stable retention in the eye for approximately 2–3 weeks without significant retinal cell layer disruption or Müller cell activation [[226], [227], [228]]. In patients with RRD, reduced intraocular HA concentration and elevated hyaluronidase activity [229] suggest that HA degradation may play a role in RRD pathogenesis, supporting the rationale for HA-based bioactive materials in retinal break repair. Ren et al. first demonstrated the clinical application of Healaflow® as a retinal patch, observing its stable adhesion and slow degradation, which prevented subretinal fluid reaccumulation [230]. Zheng et al. further optimized this system by replacing BDDE with divinyl sulfone (DVS) for linear crosslinking, achieving milder reaction conditions and better preservation of HA molecular integrity, thereby enhancing mechanical support [231].

Another commercial HA-based hydrogel, Seprafilm®, composed of sodium HA and carboxymethylcellulose (CMC), transitions into a gel upon hydration, adhering to tissue surfaces via physical adsorption to form a barrier [33,35]. Haruta et al. reported long-term follow-up (nine years) of four PPV patients treated with Seprafilm®, noting sustained retinal reattachment, visual improvement, and absence of severe complications, highlighting its potential in retinal repair (Fig. 7C) [232].

Although both originated as surgical adhesives, their formats diverge—Seprafilm® as a sheet-like barrier and Healaflow® as an injectable hydrogel. Seprafilm® shows notable drawbacks in retinal repair. Its sheet-like structure requires complex intraocular delivery under a dry environment, prolonging surgery and raising the risk of iatrogenic injury [33,35,232]. It is limited to small posterior breaks and degrades within one to two weeks, reducing long-term effectiveness [227,228,232]. In contrast, Healaflow®’s injectable hydrogel enables minimally invasive coverage of multiple or peripheral lesions and remains for several months, offering sustained support [230,231]. Clinical studies further report delivery-related complications with Seprafilm®, such as transient intraocular pressure rise and visual field defects, which have not been observed with Healaflow® [35,230]. Overall, Healaflow® demonstrates easier handling, broader applicability, and longer persistence, making it more advantageous in retinal surgery.

As a key ECM component, HA facilitates cell adhesion, migration, and differentiation, making it suitable for cell delivery. Yong et al. developed an HA-based hydrogel using HA and neural basal medium to deliver RPCs in a retinal degeneration mouse model, demonstrating uniform RPC distribution and expression of mature photoreceptor markers [233]. Environmental cues influencing cell phenotype and differentiation include chemical, mechanical, and electrophysiological signals. Youn et al. identified stress relaxation as a mechanical regulator of cell behavior and developed a GG/HA composite hydrogel via physical crosslinking for retinal tissue engineering [34]. By adjusting material composition, they created a microenvironment conducive to RPE adhesion without cell dispersion, validating its phenotype-preserving effects in vitro.

### 5.4 Synthetic bioactive materials for the ocular fundus

#### 5.4.1 Cyanoacrylate-based bioactive materials for the ocular fundus

Cyanoacrylate-based bioactive materials have been utilized in SB, a classic posterior approach for retinal detachment repair for over half a century due to their strong adhesive properties, particularly in reinforcing sutures in thin scleral regions and locally expanding compression zones [143]. With advancements in polymerization techniques and reduced toxicity, their applications have expanded from superficial tissues to deeper layers and from external to internal surgical procedures. In PPV, studies comparing cyanoacrylates to cryotherapy for sealing retinal breaks demonstrated that cyanoacrylate-induced chorioretinal adhesions not only form immediately but also exhibit significantly greater bonding strength [234,235].

However, cyanoacrylates exhibit excessively strong bonding strength and possess biomechanical properties that differ significantly from those of the retina, which may increase the risk of postoperative retinal tears. Furthermore, studies have reported that cyanoacrylates may induce localized toxicity in certain cases [236], particularly causing distinct retinal damage [237]. Documented adverse effects include crescent-shaped retinal tears at the injection site [142], immediate post-injection edema, and subsequent coagulative necrosis within one week [238]. In contrast, naturally derived bioactive materials exhibit superior biocompatibility, which further restricts the clinical applicability of cyanoacrylates in vitreoretinal surgery.

#### 5.4.2 Polyethylene glycol-based bioactive materials for the ocular fundus

PEG, a synthetic, inert, and water-soluble polymer, exhibits significant theoretical and clinical potential in ophthalmic applications due to its tissue compatibility, polymerizability, and optical transparency. Katagiri et al. developed WTG-127 hydrogel by incorporating methylcellulose into PEG, which conferred thermosensitivity and adjustable viscosity [239]. However, this formulation demonstrated suboptimal efficacy in sealing retinal breaks. To address this limitation, Hubschman et al. employed modified PEG derivatives with an ammonium acetate buffer system and ester functionalization, enabling tunable polymerization kinetics and degradation profiles [240]. DuraSeal™, an FDA-approved biocompatible hydrogel for dural repair in neurosurgical and spinal procedures, consists of an NHS ester-modified PEG crosslinked with amine-based components [241]. Sueda et al. investigated its feasibility in retinal detachment surgery but observed severe postoperative inflammation, leading to low reattachment rates and PVR, indicating the need for further optimization of material composition and delivery methods [242]. Building upon these findings, Barliya et al. pioneered a transscleral injection technique for DuraSeal™ in RRD,leveraging its dual mechanism of physical sealing and hydration-mediated expansion. This approach simplified surgical procedures by circumventing air-induced polymerization interference during vitrectomy while reducing complication rates [243]. Another commercially available adhesive, FocalSeal, incorporates acrylate-modified PEG termini, enabling photopolymerization under visible light. Hoshi et al. confirmed its favorable ocular biocompatibility and demonstrated successful long-term retinal reattachment in both ex vivo and in vivo models [244,245], offering a promising alternative to gas tamponade in retinal detachment repair.

## 6 Bioactive molecules in ocular adhesive bioactive materials

To promote tissue repair and functional recovery, recent ocular adhesive biomaterials are frequently loaded with bioactive molecules or engineered to incorporate cells capable of secreting such factors. Among the most representative examples are ocular growth factors and exosomes.

Growth factors are soluble proteins or peptides that activate signaling pathways through ligand–receptor interactions. Reported molecules in ophthalmic applications include anti-VEGF agents, which inhibit neovascularization, and ciliary neurotrophic factor (CNTF), which supports photoreceptor survival and maintenance. These agents are characterized by well-defined composition, clear pharmacodynamics, and established regulatory and CMC frameworks, conferring strong clinical accessibility. A CNTF-encapsulated cell implant has already gained approval, demonstrating the feasibility of sustained release for over ten years [246]. Similarly, long-acting polymeric or hydrogel-based anti-VEGF strategies are approaching clinical translation [247].

Exosomes are nanoscale lipid bilayer vesicles (typically 40–160 nm in diameter) secreted via multivesicular body (MVB) exocytosis. They carry diverse molecular cargo, including miRNAs/mRNAs, proteins, and lipids. Through receptor interactions, endocytosis, or membrane fusion, exosomes deliver these “cargo packages” into recipient cells, thereby modulating transcriptional, epigenetic, and protein networks in parallel. Their capacity for multi-cargo, multi-target regulation of immune, neural, and vascular processes—combined with engineered targeting and protection through hydrogels or microcapsules—has enabled exosomes to exhibit anti-inflammatory, antioxidant, anti-scarring, neuroprotective, and anti-angiogenic effects in models of diabetic retinopathy (DR), age-related macular degeneration (AMD), and ischemic–inflammatory injury [139,248,249]. Exosomes also display low immunogenicity and strong tissue penetration, enhancing their translational potential. For example, MSC-derived exosomes (MSC-Exos) can deliver miR-182 to modulate the TLR4/NF-κB/PI3K/Akt axis, shifting macrophages from the M1 to the M2 phenotype and improving the inflammatory milieu [250]. Exosomal miR-150-5p suppresses the MEKK3/JNK/c-Jun pathway, thereby reducing retinal ganglion cell apoptosis [251]. Furthermore, because corneal fibrosis is highly dependent on the TGF-β/Smad cascade and fibroblast-to-myofibroblast transition, exosomal delivery of miR-24-3p has been shown to downregulate CTGF and α-SMA expression, alleviating abnormal collagen deposition and fibrosis [252,253].

Despite these promising findings, exosomes present significant translational challenges due to their complex origin, high heterogeneity, and poorly defined composition. Consequently, most current studies remain at the animal or early translational stage. To date, no exosome-based products have received regulatory approval in the United States. The FDA has repeatedly issued safety alerts and warning letters, emphasizing that such products are generally regulated as drugs or biologics and therefore require IND/BLA review. Establishing standardized criteria for exosome consistency, potency assays, and quality control remains a key prerequisite for clinical translation and industrialization.

## 7 Practical guidance for ocular adhesive bioactive materials

Although ocular adhesive bioactive materials have become a rapidly growing research focus, relatively few have reached clinical use; accordingly, we emphasize regulatory pathways and offers practical guidance to facilitate compliant clinical translation.

In the United States, ocular adhesives and sealants designed purely for sealing or closure of ocular tissues are typically classified as Class III devices, necessitating Premarket Approval (PMA) [254]. This classification is in line with FDA's requirements for medical devices intended for use in sensitive areas like the eye, where risks are significant, and the performance must be rigorously validated. For instance, products like the ReSure®, indicated for managing clear-corneal incision leaks during cataract surgery, have set the precedent, showing superior outcomes in early postoperative leak rates when compared to traditional suturing. Such sealants are approved based on leak prevention as a primary endpoint. When the device is intended for bioactive or drug-loaded applications (e.g., antibiotics or growth factors), it may be classified as a combination product, which triggers a different regulatory pathway. This would involve coordination between FDA's Center for Devices and Radiological Health (CDRH), Center for Drug Evaluation and Research (CDER), or Center for Biologics Evaluation and Research (CBER), depending on the Primary Mode of Action (PMOA). Therefore, an early Q-Submission (Q-Sub) to FDA is recommended to align the regulatory expectations and ensure clarity regarding the combination product classification [255].

In the EU, the European Medicines Agency (EMA) does not directly approve devices, but the Notified Body (NB) is responsible for Conformité Européenne (CE) certification under the Medical Device Regulation (MDR, EU 2017/745) [256]. When the device includes human- or animal-derived components or pharmacological ingredients, EMA is consulted for scientific advice or approval, particularly when the device contains substances that could be classified under Rule 14 of MDR, which applies to devices with medicinal substances that perform an ancillary action. If the product is bioactive, such as antimicrobial or regenerative, it would likely fall under Class III and require consultation with EMA or a relevant national health authority. In this context, early communication with the NB or EMA is critical to align on classification and regulatory expectations.

For the research and regulatory teams, the first step is to clearly define the product's intended use and target indication (e.g., sealing corneal incisions or treating small perforations). This is crucial as it directly determines the regulatory classification, clinical endpoints, and appropriate controls. When the device claims sealing or leak prevention without any additional biological claims, this typically aligns with existing FDA/PMA precedents like the ReSure® Sealant, where early postoperative leak rates are the primary endpoint. If the device includes bioactive components, it is likely to be classified as a combination product, requiring comprehensive chemistry, manufacturing, and controls (CMC) data, including pharmacokinetics, release dynamics, and stability, as well as nonclinical and clinical data. In this case, early submission for Q-Sub or consultation with the FDA is advisable to confirm the regulatory pathway and ensure clarity on testing requirements [257].

Several systems that have entered clinical or pre-clinical use delineate reproducible routes to translation. In the anterior segment, CTAs and fibrin glues have long-standing clinical footprints [258], while ReSure® codified a pathway for sealing clear corneal incisions within a defined intraoperative window [161]. In the posterior segment, the crosslinked HA injectable Healaflow® [230] and the HA/CMC sheet Seprafilm® [232] illustrate distinct trade-offs in coverage, residence, and delivery. PEG-derived hydrogels such as DuraSeal™ [243] and FocalSeal® [244,245] continue to underpin animal and early retinal repair studies.

Taken together, the evidence suggests that successful translational pathways often share several intrinsic characteristics. First, validation is conducted around clinical scenarios and endpoints that are clearly defined and quantifiable. For instance, the ocular surface focused on negative Seidel tests and leakage or burst pressure as primary outcomes, while the posterior segment emphasized hole closure with single-operation reattachment as key indicators [259]. Second, priority is given to chemical agents and processes with established safety profiles and scalability in ophthalmology or related fields, with further reinforcement of wet-state stability and durability through low-swelling dual-network or composite structures. Third, a high degree of compatibility with existing surgical techniques is highlighted, including the use of two-component, ready-to-apply systems, operative windows of only several tens of seconds, and reliable conformity to curved surface [260]. Finally, during the early indication phase, the primary function lies in providing mechanical sealing or physical barriers, after which drug delivery and regenerative modules may be progressively integrated within a compliant regulatory framework.

For the clinical teams, when selecting adhesive materials, it is essential to consider the risk of the intended use. If leakage (Seidel positive) is identified during surgery, and a temporary dry field can be obtained, an approved ocular adhesive gel can be used for sealing. However, for profuse leakage or inability to achieve dryness—or for high-risk surgical sites—traditional suturing or a combination of sutures and sealant should be considered. Before and after the application of an ocular adhesive, it is crucial to confirm the leak status through standardized Seidel testing to ensure proper closure and prevent postoperative complications such as leakage or infection.

In clinical practice, the balance between adhesion and bioactivity depends on context. Key factors include the anatomical site (anterior vs. posterior segment), defect size and depth, urgency, optical and mechanical matching, wet-interface shear, and the need for drug delivery or regeneration.

In emergencies such as perforation or active leak, strength and speed are decisive. CTA remains a mainstay for small perforations because it polymerizes rapidly and provides immediate tectonic support. However, its histotoxicity, risks of inflammation, ocular hypertension, and neovascularization, and the frequent need for re-application mean CTA is best used as a bridge to definitive repair [11]. For corneal stromal defect reconstruction—where transparency, integration, and regeneration drive long-term outcomes—collagen/gelatin- and PEG-based materials better align optical clarity, mechanical compatibility, and tissue integration on the persistently wet corneal surface.

In the posterior segment, mechanical demands on adhesion are usually lower, but drug-delivery inefficiency remains the major unmet need. Gelatin-, and HA-based platforms are advancing toward instant sealing with sustained release, aiming to patch retinal breaks while supporting durable functional recovery [233,261].

In both the FDA and EU regulatory frameworks, early engagement with the relevant regulatory bodies, whether it is FDA's Q-Sub or EMA's consultation processes, is essential to ensure alignment on the appropriate regulatory classification, clinical design, and endpoint strategies. This proactive approach can significantly streamline the path to approval and market entry for ocular bioactive adhesives.

## 8 Challenges and future prospects

According to the criteria proposed by Oelkler and Grinstaff, an ideal ocular surface adhesive should meet the following benchmarks: leak pressure (>80 mmHg), crosslinking/gelation time (<30 s), mechanical modulus (5–200 kPa), swelling (<200 %), diffusion coefficient (>2 × 10^−7^ cm^2^/s), refractive index (1.32–1.40), cytotoxicity compliant with ISO standards, adhesion strength (>0.1 kPa), viscosity (5–100 cP), degradation time (1 week–6 months), and wound residence time (1 day–6 months) [262]. Contemporary formulations typically satisfy most of these requirements; consequently, current research emphasizes further optimization of structure and function. However, the growing diversity of clinical indications and device concepts suggests that these benchmarks should not be regarded as immutable endpoints, but rather as a starting point for indication-specific refinement.

As our understanding of ocular surface adhesives has matured, the Oelker–Grinstaff benchmarks are best viewed as a starting framework rather than hard limits [262]. For example, swelling should be treated as application-dependent: modest, controlled swelling can help plug microleaks and improve interfacial contact. However, excessive swelling—especially in large defects or along the visual axis—risks optical aberrations and delayed epithelialization. Contemporary designs therefore bias toward “minimal but sufficient” swelling; for on-axis indications, several groups deliberately target ≤20–30 % swelling ex vivo to preserve optical quality, whereas more latitude may be acceptable peripherally [94]. In addition, the ocular surface imposes repetitive, blink-driven shear and tear-flow exposure, so fatigue resistance under cyclic shear is essential. Blink mechanics produce very high shear rates; combined with the tear film's shear-thinning viscosity, this translates to shear stresses on the order of several to hundreds of pascals, depending on instantaneous viscosity and blink speed [[263], [264], [265], [266], [267]]. Finally, degradation should be synchronized to tissue repair timelines: days for epithelial sealing, weeks–months for corneal stromal remodeling, and months–years for posterior segment repair strategies. Even when these physicochemical and mechanical parameters are carefully tuned, current materials remain insufficient to meet the multifactorial demands of real-world ocular pathology.

At present, adhesive bioactive materials for ophthalmic applications still face several limitations. Structurally, many of these materials are suitable only for repairing small incisions or minor defects, but they fail to achieve complete restoration in cases of extensive or severe tissue damage. Functionally, ocular injuries and diseases are often caused by multiple factors; however, some adhesive materials exhibit relatively limited functions. They can bond separated tissues but lack the capacity to simultaneously provide anti-inflammatory effects, promote healing, and prevent scar formation. In the following section, we discuss several innovative strategies in structural and functional design.

For corneal applications, biomaterials must achieve mechanical matching and optical transparency while preserving native corneal cell phenotypes, which is crucial for durable tissue repair and functional recovery. Beyond continued compositional refinement and biomimetic strategies such as collagen alignment and fibril cross-linking, maintaining the ordered lamellar–microfibrillar architecture remains a central design target for both optics and mechanics. Although current in situ–curing adhesives remain constrained in their ability to faithfully recapitulate the native corneal ultrastructure, adhesive patches designed to substitute for or guide corneal repair could deliberately integrate structure-shaping techniques during fabrication to confer enhanced structural and functional biomimicry.

Several approaches have been explored to replicate corneal ultrastructure, including direct retention of decellularized native scaffolds, electrically triggered “directed” self-assembly, small-molecule modulation, tissue-scale replica molding, and 3D bioprinting. Directly preserving a decellularized corneal stroma as the principal load-bearing and optical framework is technically straightforward but may be limited by donor availability and carries risks of disease transmission and immune rejection [268].

In electro-triggered self-assembly, applying a cathodic field to acidic collagen solutions induces rapid, localized microfibril assembly near the cathode; the resulting lamellae exhibit greater transparency and lower haze than products of conventional thermodynamic assembly, and electrode geometry/current density can be used to tune curvature and thickness to match refractive power [269].

As a representative small-molecule route, cyclodextrins reversibly complex with hydrophobic collagen residues (e.g., tyrosine), suppressing excessive coarsening and guiding ordered fiber/lamellar growth; scaffolds produced in this manner display mechanics closer to native cornea and improved suturability [270].

Tissue-level templating (replica molding) uses the hierarchical structure of natural tissue as a template: a curable precursor is infused into the micro/nanopores and interfibrillar spaces of native cornea, polymerized in situ, and the template is then removed to yield an inverse replica; subsequent back-filling with the target matrix/cells produces a high-fidelity positive replica. This strategy enables accurate reproduction of hierarchical features while relying on accessible sources, for example, xenogeneic decellularized tissue as the template and humanization via perfusion of surgical-waste human corneal matrix [114].

3D bioprinting can fabricate scaffolds that closely mimic corneal architecture and, through digital design and automation, shorten production time and reduce material waste [271]; embedding cells and bioactive agents (e.g., growth factors, antimicrobials) directly into bioinks may accelerate repair and reduce postoperative infection risk. Although 3D-printed corneal substitutes remain at the preclinical stage [272], convergence with emerging technologies such as artificial intelligence could enable precision diagnosis and personalized implants; artificial neural networks may enhance automation, minimize operator-induced variability, and tailor constructs to individual anatomy and disease status.

To address low ocular bioavailability and the difficulty of posterior-segment delivery, ocular bioactive materials are being integrated with stimuli-responsive drug delivery. Reported triggers include pH [273], enzymes [274], ROS [275], light [276], temperature [277], ionic strength [278], mechanical cues [279] and ultrasound/electromagnetic fields [280]. Such stimuli can modulate material properties and behaviors—e.g., sol–gel transitions and release kinetics—enabling spatiotemporal programming. In this way, adhesive biomaterials are engineered into multifunctional platforms that combine robust adhesion with anti-inflammatory, antioxidant, and pro-regenerative functions suited to the complex ocular environment.

**Fig. 8:**
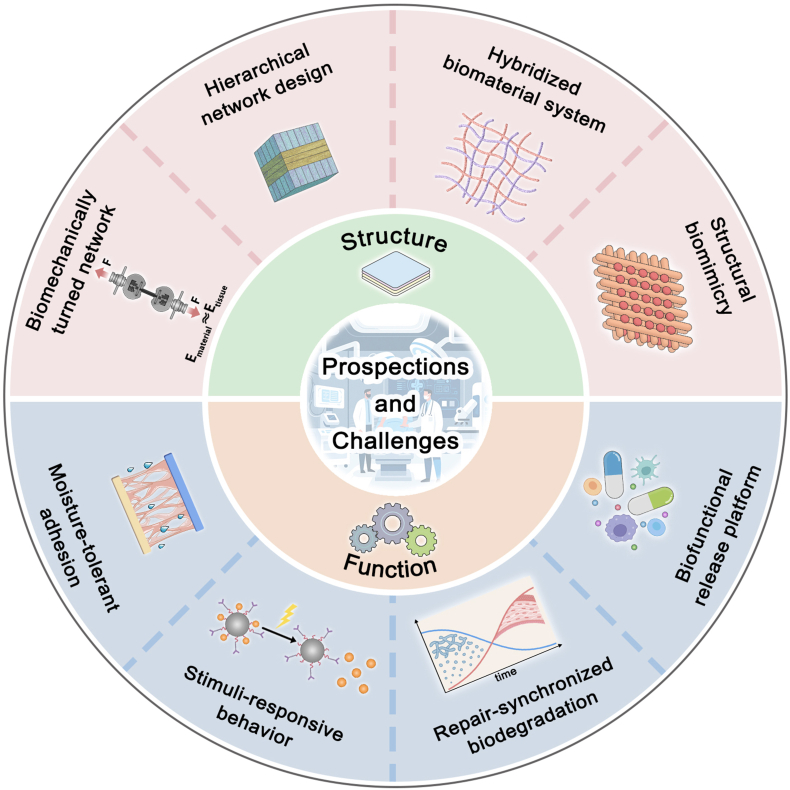
Prospections and challenges of adhesive bioactive materials in ophthalmology.

In summary, while ocular adhesive bioactive materials represent a transformative approach for ophthalmic therapy, their clinical translation continues to encounter several challenges (Fig. 8). Structurally, achieving biomechanical compatibility between the adhesive material and surrounding ocular tissues remains paramount. Key innovations in material design include hierarchical network architectures, bioinspired material strategies, and multicomponent hybrid systems. Functionally, maintaining strong and stable adhesion under the eye's moist conditions is essential to ensure effective performance. Key directions in intelligent material development encompass stimuli-responsive behaviors, degradation rates aligned with tissue healing, and the integration of bioactive agents. These features collectively bring ocular adhesive bioactive materials closer to clinical translation, fulfilling the specialized needs of ocular tissue repair and regeneration.

## 9 Conclusions

Ocular adhesive bioactive materials have become multifunctional platforms that can replace sutures, promote tissue regeneration, and enable controlled delivery of bioactive molecules. Innovations in naturally derived and synthetic materials have significantly enhanced their mechanical, optical, and biological performance. Despite substantial progress, challenges persist, particularly in achieving strong, long-lasting adhesion in wet ocular environments and replicating the biomechanical and optical properties of native tissues. Future research should emphasize biomimetic design, intelligent responsiveness to stimuli, and integration with regenerative therapies such as growth factors and exosomes. Clinical translation will require rigorous, adequately powered studies and standardized preparation protocols. With continued interdisciplinary efforts, bioactive adhesive materials will play a transformative role in minimally invasive, patient-tailored ocular therapies.

## CRediT authorship contribution statement

**Yifei Niu:** Writing – original draft, Conceptualization. **Saiqun Li:** Writing – original draft, Formal analysis. **Fei Yu:** Writing – original draft, Formal analysis. **Xuan Zhao:** Writing – review & editing, Supervision, Funding acquisition. **Jin Yuan:** Writing – review & editing, Supervision, Funding acquisition.

## Ethics approval and consent to participate

This review article does not involve human participants, human data, or animal experiments. Therefore, ethics approval and consent to participate are not applicable.

## Funding source

This work was supported by the 10.13039/501100001809National Natural Science Foundation of China (82230033 and 82171015), the 10.13039/501100003453Natural Science Foundation of Guangdong Province (No. 2024A1515011101).

## Declaration of competing interest

Jin Yuan is an editorial board member for Bioactive Materials and was not involved in the editorial review or the decision to publish this article. All authors declare that there are no competing interests.

## Abbreviations

AHTL: DL-N-acetyl homocysteine thiolactone
AIDS: Acquired immunodeficiency syndrome
AlgMA: Methacrylated alginate
AMD: Age-related macular degeneration
APC: Autologous platelet concentrate
APCS: Acellular porcine corneal stroma
BDDE: 1,4-Butanediol diglycidyl ether
CBER: Center for Biologics Evaluation and Research
CDER: Center for Drug Evaluation and Research
CDRH: Center for Devices and Radiological Health
CE: Conformité Européenne
CEC: Corneal endothelial cell
CMC: Carboxymethylcellulose
CMHA-S: Thiolated carboxymethyl hyaluronic acid
CNTF: Ciliary neurotrophic factor
CS: Chitosan
CSNPs: Chitosan nanoparticles
CTAs: Cyanoacrylate tissue adhesives
CV: Collagen vitrigel
DA: Dopamine
DMA: Dopamine methacrylamide
DMEK: Descemet's membrane endothelial keratoplasty
DR: Diabetic retinopathy
DVS: Divinyl sulfone
ECM: Extracellular matrix
EDTA: Ethylenediaminetetraacetic acid
EMA: European Medicines Agency
F127-DA: Pluronic F127 diacrylate
FDA: Food and Drug Administration
G-AA: Acrylated gelatin
GG: Gellan gum
GelMA: Gelatin methacryloyl (methacrylate)
GMA: Glycidyl methacrylate
G-SH: Thiolated gelatin
HA: Hyaluronic acid
HA-NB: Nitrobenzyl-modified hyaluronic acid
HAGM: Hyaluronic acid-glyceryl methacrylate
HAMA-DOPA: Dopamine-modified methacrylated hyaluronic acid
hCECM: Human corneal extracellular matrix
HPA: Hydroxyphenylpropionic acid
hPL: Human platelet lysate
ICG: Indocyanine green
ICRS: Intrastromal corneal ring segment
IOP: Intraocular pressure
IPNs: Interpenetrating polymer networks
iPSC-MSC: Induced pluripotent stem cell-derived mesenchymal stem cell
LAP: Phenyl-2,4,6-trimethylbenzoylphosphinate (photoinitiator)
LASIK: Laser-assisted in situ keratomileusis
LESCs: Limbal epithelial stem cells
MA: Methacrylic anhydride
MA-HA: Methacrylated hyaluronic acid
MD: Macular detachment
MDR: Medical Device Regulation
MHs: Macular holes
MSC-Exos: Mesenchymal stem cell-derived exosomes
mTG: Microbial transglutaminase
MVB: Multivesicular body
NB: Notified body
NHS: N-hydroxysuccinimide
OCT: Optical coherence tomography
ODex: Oxidized dextran
OGG: Oxidized guar gum
OHA: Oxidized hyaluronic acid
PA: 4-Pentenoate (4-pentenoic acid moiety)
PAMAM: Poly(amidoamine)
PCL: Polycaprolactone
PDA: Polydopamine
PDGF: Platelet-derived growth factor
PEG: Polyethylene glycol
PEGDA: Polyethylene glycol diacrylate
PLA2: Phospholipase A2
PMA: Premarket approval
PMOA: Primary mode of action
PPV: Pars plana vitrectomy
PVR: Proliferative vitreoretinopathy
Q-Sub: Q-Submission
RFP: Riboflavin phosphate
RGC: Retinal ganglion cell
RHC: Recombinant human collagen
RPC: Retinal progenitor/precursor cell
RPE: Retinal pigment epithelium
RRD: Rhegmatogenous retinal detachment
ROS: Reactive oxygen species
SB: Scleral buckling
SF: Silk fibroin
SH-HA: Thiolated hyaluronic acid
sIPN: Semi-interpenetrating polymer network
SPAAC: Strain-promoted azide-alkyne cycloaddition
TEA: Triethanolamine
TGF-β: Transforming growth factor-beta
TGase: Transglutaminase
UV: Ultraviolet
VC: N-vinyl caprolactam
