# Supplementary material for: Adhesive bioactive materials in ocular applications: Toward smart, regenerative, and minimally invasive therapies
Source: Bioact Mater. 2025 Dec 12;58:303–30. doi: 10.1016/j.bioactmat.2025.12.004 (PMC12765266; doi:10.1016/j.bioactmat.2025.12.004)
Supplement: Multimedia component 1 [file mmc1.docx]

**Supplementary file**

**Table S1. Main components and advantages of adhesive bioactive materials in ocular applications**

| Application site | Material Type | Material Name | Last Corresponding Author | Main Components | Main advantages |
| --- | --- | --- | --- | --- | --- |
| Ocular surface | Blood-derived bioactive materials | Human platelet lysate-based biomaterial[62] | Gerard Sutton | Platelet lysate, human fibrinogen, and human thrombin | Accelerates healing and reduces pain |
|  |  | Temperature-controlled laser soldering with bovine albumin[64] | Dan D. Gaton | Bovine albumin | Less neovascularization, complete epidermal regeneration and shorter procedure time |
|  | Collagen-based bioactive materials | Col/DAD hydrogel[68] | Xin Chen | Dextran and collagen | Thermostable collagen hydrogel with high mechanical strength |
|  |  | Collagen-Dextran-GD nanoparticles hydrogel[69] | Manikantan Syamala Kiran | Dextran, collagen and gadolinium oxide | Anti-angiogenic and anti-inflammatory |
|  |  | Hybrid nanostructured gadolinium oxide-collagen-dextran polymeric hydrogel[65] | Sun Lingyun | Recombinant human collagen modified with methacrylic anhydride | The physicochemical properties are similar to natural cornea |
|  |  | HA- and collagen-based interpenetrating polymer network (IPN)[66,72] | David Myung | Collagen and hyaluronic acid (HA) | Dual collagen-HA networks via SPAAC/Michael addition crosslinking forming transparent hydrogels |
|  |  | SPAAC gel[39] | David Myung | Azide-polyethylene glycollagen (PEG)-collagen and dibenzocyclooctyne-collagen | Promotes corneal epithelial cell proliferation and supports stromal cell spreading |
|  |  | Injectable collagen hydrogel based on covalently modified acetyl thiol collagen[70] | Ayan Samanta | Collagen and polyethylene glycol | Natural cornea-matched tunable mechanics and Fibrin glue-equivalent tissue adhesion |
|  |  | ColMA[73] | Hongwei Ouyang | Methacryloylated collagen (ColMA) | Complete biodegradation and regeneration, controlled and non-inflammatory degradation process |
|  | Gelatin-based bioactive materials | PDA@GelMA[92] | Paul S. Weiss | Dopamine (DA, Gelatin methacryloyl (GelMA) prepolymer | Improved surface roughness and adhesion |
|  |  | GelCORE[93] | Nasim Annabi | GelMA, Type 2 Eosin Y, triethanolamine (TEA) and N-vinyl caprolactam (VC) | Avoids ultraviolet (UV) light cross-linking, reducing potential ocular photochemical toxicity. |
|  |  | GelPatch[94] | Nasim Annabi​ | GelMA and glyceryl methacrylate-ylated HA (HAGM) | Strong adhesion, low swelling ratio and suitable modulus of elasticity |
|  |  | ECM-Like Adhesive Hydrogel for large corneal defects[95] | Qingjun Zhou | GelMA and N-(2-aminoethyl)-4-(4-(hydroxymethyl)-2-methoxy-5-nitrosophenoxy) butanamide-modified HA (HA-NB) | High light transmittance, good adhesion to wet tissues, rapid gelation upon light exposure |
|  |  | GMO[96] | Li Ren | GelMA and oxidized hyaluronic acid (OHA) | Outstanding adhesive and mechanical properties |
|  |  | GelMA/SF[97] | Ayça Bal-Öztürk | GelMA and silk fibroin (SF) | Cornea-matched transparency, enhanced adhesion and optimized ocular safety |
|  |  | Photocurable bioadhesive hydrogel composed of GelMA and ODex[98] | Jin Yuan | GelMA and oxidized dextran (ODex) | Double-network photocurable hydrogel with high adhesive strength |
|  |  | Dual covalent cross-linking hydrogel bioadhesive[21] | Li Ren | Acrylated gelatin(G-AA), thiolated gelatin(G-SH) and dextran | Enhanced long-term wet adhesion |
|  |  | Gel/DMA[99] | Wei Chen | GelMA and dopamine methacrylamide (DMA) | Enhanced tissue adhesion, ROS scavenging and anti-inflammatory properties |
|  |  | iSK@Gel[100] | Luyang Yu | GelMA and human amniotic epithelial stem cells (hAESCs) | Mimick the structural and mechanical properties of the corneal stroma |
|  |  | “T.E.S.T.” hydrogel[101] | Xingtao Zhou | GelMA, polyacrylic acid F127 diacrylate (F127DA), aldolized polyacrylic acid F127 (AF127), and collagen type I | High strength, transparency, bioadhesion, slow degradation, tissue integration |
|  |  | Injectable, UV-curable gelatin system[102] | Timothy C. Hughes​ | Acryloylated gelatin (GE-AA) and mercaptoethylated gelatin (GE-SH) crosslinked by a thiol-acrylating reaction | Tunable properties, high transparency, biodegradability, and cell compatibility |
|  |  | GELGYM[103] | Miguel Gonzalez-Andrades | Gelatin and glyceryl methacrylate (GMA) | Improved elasticity and biomimetic properties |
|  | Decellularized corneal stroma matrix-based bioactive materials | Dual-crosslinked regenerative hydrogel[20] | Ying Bai | Decellularized porcine corneal ECM, methacrylate anhydride (MA) and chemical cross-linking agents | Strong tissue adhesion and long-term stability |
|  |  | GelCodE[109] | Dong-Woo Cho | Gelatin and cornea-derived extracellular matrix (ECM) | Low contact angle, high adhesion and high plasticity |
|  |  | APCS-gel[110] | Mark I. Rosenblatt | Acellular porcine corneal stroma (APCS) | High transparency and rapid gelation but weak mechanical properties |
|  |  | Human cornea-derived ECM hydrogel[111] | Falguni Pati | ECM of non-transplant-grade human corneas | Injectable, biocompatible and rapid epithelialization |
|  |  | LC-COMatrix[112] | Ali R Djalilian | Decellularized porcine corneal ECM and methacrylate anhydride (MA) | Visible light curable, high transparency and tunable mechanics |
|  |  | IonBAH[113] | Weiyun Shi | Peptide-modified alginate, transglutaminase, porcine corneal stromal solution and calcium ions adhesion | Dual-network stability, strong tissue adhesion, and large-size defect applicability |
|  |  | Humanized Corneal Stroma-like adhesive Patch (HCSP)[114] | Weiyun Shi | Human-derived corneal extracellular matrix (hCECM), polyethylene glycol diacrylate (PEGDA) and GelMA | Biomimetic lamellar architecture and enhanced epithelial regeneration |
|  | HA-based bioactive materials | In situ-forming HA hydrogel[40] | David Myung | MA-HA and sulfhydrylated HA (SH-HA) | Delayed gelation to avoid direct light exposure to the tissue |
|  |  | User-demand fast-curable ocular glues[22] | Seung Yun Yang | HA, MA and 4-pentenoic acid (PA) | Dual crosslinking structure with fast start and late reinforcement |
|  |  | Photocurable and Temperature-Sensitive Bioadhesive Hydrogels[38] | Jin Yuan | Polyether F127 diacrylate (F127DA) and dopamine-modified HA methacrylate (HAMA-DOPA) | Improved adhesion strength, stability and high light transmission |
|  |  | Supramolecular HA hydrogels[23] | David Myung | HA-cyclodextrin (HA-CD) and HA-adamantane (HA-Ad) | Supramolecular crosslinking and shear-thinning behavior |
|  |  | OcuPair[124,125] | Rangaramanujam M. Kannan | HA and methacrylated, hydroxyl PAMAM dendrimer | Superior strength, intraocular pressure resistance, and ease of application/removal |
|  | Dextran-based bioactive materials | Chemically Defined Bioadhesive[127] | Takahiro Nakamura | Dextran and ε-poly(L-lysine) | Biodegradable, transparent and flexible hydrogel with potential bacteriostatic properties |
|  |  | T-AlgDD[128] | Juan Wang | Dextran, alginate methacryloyl, cryopolymerization of amine-terminated polyamidoamine (PAMAM) and Ca²⁺ | Triple-crosslinked double-network structure with enhanced mechanical and adhesive performance |
|  | Chitosan-based bioactive materials | CS-HYA[131] | Li Ren | Chitosan (CS) and HA | Enhanced surface hydrophilicity and improved cytocompatibility |
|  |  | A multi-function biomacromolecule chitosan–collagen composite membrane[132] | Li Ren | CS, collagen and 1-ethyl-3-(3-dimethylaminopropyl) carbodiimide | Enhanced tensile strength and improved hydrophilicity |
|  |  | DC-collagen/Chi film[133] | Petr Saha | CS, collagen, tannic acid and genipin | Dual crosslinking for enhanced mechanical properties and less toxity |
|  |  | Laser-activated chitosan adhesive film[134,135] | Stephanie Louise Watson | CS and Indocyanine Green | Laser-activated rapid sealing |
|  |  | Photo-Crosslinked Urocanic-Acid-Modified Chitosan[136] | Tai-Horng Young | CS, urocanic acid and methylene blue | Adjustable curing time with red light, high biocompatibility and low toxicity |
|  |  | Collagen/HA-based nanobrous membrane[137] | Zhongru Gou | CS, collagen, HA and polyethylene oxide | Anti-fibrotic effect compared to human amniotic membrane |
|  |  | CSNP/CS /PCL composite scaffolds[138] | Hassan Niknejad | CS, polycaprolactone (PCL) and chitosan nanoparticles (CSNPs) | Transparent and biocompatible scaffold that is suitable for corneal endothelial regeneration |
|  |  | Thermosensitive CS-based hydrogels[139] | Ke Yao | CS and exosomes from induced pluripotent stem cell-derived mesenchymal stem cell (iPSC-MSCs-Exos) | miR-432-5p-mediated ECM remodeling and collagen downregulation |
|  |  | MSC-Exos@OGG/CMCS hydrogel[140] | Meiyan Li | Carboxymethyl chitosan (CMCS), oxidized guar gum (OGG) and mesenchymal stem cell-derived exosomes (MSC-Exos) | Self-healing and injectable hydrogel with anti-inflammatory and anti-scarring effects |
|  | Cyanoacrylate-based bioactive materials | Cyanoacrylate tissue adhesives[141–145] | N/A | Alkyl cyanoacrylate | High mechanical strength but poor compatibility |
|  | PEG-based bioactive materials | ReSure®[155–162] | N/A | Four-armed PEG prepolymer capped with N-hydroxysuccinimide and a tri-lysine cross-linker | Hydrophilic and non-toxic |
|  |  | Polysaccharide-based tissue adhesive[163,164] | George K. Kodokian | Multi-arm PEG amine and dextran | Biocompatible and non-cytotoxic |
|  |  | Chondroitin sulfate-PEG[165] | Jennifer H. Elisseeff | PEG and chondroitin sulfate | High mechanical strength and pressure resistance |
|  |  | Chondroitin sulfate-PEG+CV[166] | Jennifer H. Elisseeff | PEG, chondroitin sulfate and collagen vitrigel (CV) | Enhanced wound sealing capacity |
|  |  | EPPH (Janus hydrogel)[167] | Yibo Yu | PEG, oxidized heparin (PPH) and thiolated ε-poly-L-lysine (EPL-SH) | Anti-fibrosis and broad-spectrum antibacterial activity |
|  |  | LiQD Cornea[168–170] | May Griffith | PEG and collagen-like peptide (CLP) | Cost-effective alternative to donor grafts, supports corneal regeneration and nerve ingrowth |
|  |  | PEG-collagen hydrogel[36,171,172] | David Myung | Multi-armed PEG-N-hydroxysuccinimide (NHS) and bovine collagen type I protein | Mechanically tunable and optically clear hydrogel supporting epithelial regeneration without fibrosis |
|  |  | Fiber-reinforced GelMA hydrogel[173] | Shengli Mi | PEG, poly caprolactone (PCL) and GelMA | Orthogonally aligned fiber-guided hydrogel that supports keratocyte phenotype and ECM alignment |
|  |  | GelMA–HAGM–PEGDA hydrogel patch[174] | Reza Dana | PEGDA, GelMA and HA glyceryl ether methacrylate (HAGM) | Light-activated in situ crosslinking and effective sealing of complex full-thickness ocular wounds |
|  |  | PEG-modified SF membrane[175] | Haiwei Xu | PEG and silk fibroin (SF) | Effective restoration of limbal stem cell deficiency long-term integration and transparency in vivo |
|  |  | PEG-based Hydrogel Films[176] | Greg G. Qiao | PEG, sebacoyl chloride and PCL | Excellent nutrient permeability for CEC viability and suitable for minimally invasive procedures |
|  |  | FXa-degradable biohybrid hydrogel[177] | Carsten Werner | PEG and chondroitin sulfate | Enzyme-triggered cell sheet release that supports functional corneal endothelial tissue formation |
| Ocular fundus | Fibrin-based bioactive materials | Fibrin glue[187–190] | Sayan Basu | Fibrinogen, Thrombin, Ca²⁺ | High cytocompatibility but relative low mechanical strength |
|  | Platelet-based bioactive materials | Autologous platelet concentrate[199–209] | Jean-Francois Korobelnik | Human platelets | High anatomical closure rates in macular hole patients |
|  | Gelatin-based bioactive materials | Gelatin plugs[210] | Aref Rifai | Gelatin | Effectiveness in achieving anatomical closure |
|  |  | GelMA[211] | Fang Zhang | GelMA hydrogel with a 75% degree of substitution and 20% concentration | Rapid photo-crosslinking and strong adhesion for retinal sealing |
|  |  | Gelatin-mTG[212,213] | Satoshi Okinami | Gelatin and microbial transglutaminase (mTG) | Strong wet tissue adhesion and no retinal toxicity |
|  |  | Ge/GG/CS hydrogel[214] | Gilson Khang | Gelatin, gellan gum (GG) and CS | Electrostatically enhanced mechanical strength and porous ECM-mimetic structure |
|  |  | Gtn-HPA hydrogel[215,216] | Michael J. Young | Gelatin, hydroxyphenylpropionic acid (HPA), horseradish peroxidase (HRP) | Improved subretinal graft localization and minimized immune response |
|  |  | Gel-HA-PDA​[217] | Ping Gu | Gelatin-SH, HAMA and polydopamine (PDA) | Mussel-inspired adhesion enhancement, enhanced RPC proliferation and neuronal differentiation |
|  |  | Cur@PDA@GelCA[218] | Jiashing Yu | Gelatin, cinnamic acid, polydopamine nanoparticles (PDA) and curcumin-loaded nanoparticles | Superior efficiency in clearing ROS and improvement in survival rate of retinal ganglion cells (RGC). |
|  | HA-based bioactive material | Healaflow®[226–230] | Xiaorong Li | HA and 1,4-butanediol diglycidyl | Effective retinal break patching |
|  |  | HA-engineered hydrogel[231] | Xiaorong Li | HA and divinylsulfone (DVS) | Linearly crosslinked HA hydrogel with high biocompatibility and mechanical stability |
|  |  | Seprafilm®[33,35,232] | R Yamakawa | HA and carboxymethylcellulose (CMC) | Strong adhesion to wet retinal surface, non-inflammatory and electrophysiologically safe |
|  |  | HA hydrogel[233] | Michael J. Young | HyStem (a commercially modified HA), Extralink (a multi-thiol polymer) and neurobasal medium | Tunable stiffness for retinal tissue matching supporting stem cell self-renewal and differentiation |
|  |  | GG/HA hydrogel[34] | Gilson Khang | HA and GG | Stress-relaxing matrix enhancing RPE-specific gene expression |
|  | Cyanoacrylate-based bioactive materials | Histoacryl[143,237] | Brooks W. McCuen II | Butyl-2-cyanoacrylate | High adhesive strength but marked cytotoxicity |
|  | PEG-based bioactive material | WTG-127[239] | Masahiko Usui | PEG and methylcellulose | Thermally responsive gelation but short-term stability |
|  |  | A PEG-derived retinal patch[240] | Ben Glasgow | PEG-derived polymer and methylcellulose | Customizable degradation and polymerization kinetics |
|  |  | DuraSeal™[241–243] | Tatsuo Hirose | NHS-modified PEG and amine component | Dual adhesion mechanism but strong postoperative inflammation |
|  |  | FocalSeal[244,245] | Hoshi et al. | Acrylate-modified PEG | Strong adhesion |
